# The Transcriptional Responses and Metabolic Consequences of Acclimation to Elevated Light Exposure in Grapevine Berries

**DOI:** 10.3389/fpls.2017.01261

**Published:** 2017-07-20

**Authors:** Kari du Plessis, Philip R. Young, Hans A. Eyéghé-Bickong, Melané A. Vivier

**Affiliations:** ^1^Institute for Wine Biotechnology, Department of Viticulture and Oenology, Stellenbosch University Stellenbosch, South Africa; ^2^Institute for Grape and Wine Sciences, Department of Viticulture and Oenology, Stellenbosch University Stellenbosch, South Africa

**Keywords:** grape, microclimate, photosynthesis, RNAseq analysis, acclimation to stress

## Abstract

An increasing number of field studies that focus on grapevine berry development and ripening implement systems biology approaches; the results are highlighting not only the intricacies of the developmental programming/reprogramming that occurs, but also the complexity of how profoundly the microclimate influences the metabolism of the berry throughout the different stages of development. In a previous study we confirmed that a leaf removal treatment to Sauvignon Blanc grapes, grown in a highly characterized vineyard, primarily affected the level of light exposure to the berries throughout their development. A full transcriptomic analysis of berries from this model vineyard details the underlying molecular responses of the berries in reaction to the exposure and show how the berries acclimated to the imposing light stress. Gene expression involved in the protection of the photosynthetic machinery through rapid protein-turnover and the expression of photoprotective flavonoid compounds were most significantly affected in green berries. Overall, the transcriptome analysis showed that the berries implemented multiple stress-mitigation strategies in parallel and metabolite analysis was used to support the main findings. Combining the transcriptome data and amino acid profiling provided evidence that amino acid catabolism probably contributed to the mitigation of a likely energetic deficit created by the upregulation of (energetically) costly stress defense mechanisms. Furthermore, the rapid turnover of essential proteins involved in the maintenance of primary metabolism and growth in the photosynthetically active grapes appeared to provide precursors for the production of protective secondary metabolites such as apocarotenoids and flavonols in the ripening stages of the berries. Taken together, these results confirmed that the green grape berries responded to light stress much like other vegetative organs and were able to acclimate to the increased exposure, managing their metabolism and energy requirements to sustain the developmental cycle toward ripening. The typical metabolic consequences of leaf removal on grape berries can therefore now be linked to increased light exposure through mechanisms of photoprotection in green berries that leads toward acclimation responses that remain intact until ripening.

## Introduction

Plants show remarkable adaptability to environmental factors and/or stresses to ultimately ensure that their core metabolic functions are maintained. Although these aspects have been intensively studied in model plants under controlled conditions to establish the basic principles and underlying pathways, as technologies developed, our ability to study and understand crop plants in their cultivated natural environments are yielding important information regarding the processes of stress protection and specifically the concept of acclimation.

In plant biology, stress is generically defined as any unfavorable conditions that affect metabolism, growth and/or development (Lichtenthaler and Burkart, 1996). The relative tolerance/sensitivity of the affected plant subsequently determines if a stress factor will have a positive (eustress) or negative (distress) outcome (Kranner et al., 2010). Acclimation refers to the short-term responses of plants to adapt to unfavorable (stress) factors in their immediate environment (Lichtenthaler and Burkart, 1996; Lichtenthaler, 1998); whereas adaptation refers to plants' long-term survival strategy to stress factors that occurs via genetic changes such as mutations and subsequent natural selection over many generations within a population. When compared to adaptation, acclimation is a rapid response, occurs within individuals, is reversible, and does not involve any permanent genetic changes. Acclimation can involve transcriptional, metabolic and/or physiological responses to improve the performance and survival of the individual to the stress. The ability of biennial plants (e.g., onions, cabbages, and carrots) to survive winter (Andrews, 1996) and the accumulation of phenolic compounds in response to increased light exposure (Caldwell et al., 1983), are examples of acclimation to low temperature and UV-B, respectively.

In grapevine, acclimation to climatic conditions is particularly important and the plasticity of grapevine responses have been highlighted in a number of publications (overviewed recently in Kuhn et al., 2014). The transcriptomic and metabolic reprogramming occurring during grape berry development has been well studied (Zenoni et al., 2010; Sweetman et al., 2012; Palumbo et al., 2014; Pilati et al., 2014; Wong et al., 2016). Research on abiotic stress factors has focused on the dominant environmental factors either individually: temperature (Carbonell-Bejerano et al., 2013; Rienth et al., 2014), light (Wu et al., 2014; Reshef et al., 2017; Sun et al., 2017), UV (Martinez-Luscher et al., 2014; Suzuki et al., 2015; Matus, 2016), and water deficit (Ghan et al., 2015; Santo et al., 2016; Savoi et al., 2016) or collectively as terroir or vintage studies (e.g., Santo et al., 2013; Anesi et al., 2015).

Light has long been recognized as central to plant metabolism through photosynthesis, but recent studies have highlighted the importance of light as a source of information for plants (reviewed in Apel and Hirt, 2004; Eberhard et al., 2008; Li et al., 2009 and references within). In viticulture, many canopy management practices are performed to optimize light exposure to drive photosynthesis of the canopy (reviewed in Smart, 1985; Clingeleffer, 2010). Apart from leaves, other plant organs including the stems, flowers, tendrils and fruits contain functional chloroplasts, and are capable of photosynthesis (reviewed in Blanke and Lenz, 1989). The conditions under which photosynthesis occurs in these non-foliar organs, however, are markedly different to their foliar counterparts. In fruits, for example, the gradual disappearance of stomata and/or the development of an impermeable waxy cuticle during development results in an internal environment that is characterized by high CO_2_ and low O_2_ (hypoxic) levels (Blanke and Leyhe, 1987, 1988; Kyzeridou et al., 2015). Decreased photosynthesis in green fruits can be attributed to these physical/anatomical features, rather than a decrease in the photosystems. Kyzeridou et al. (2015) demonstrated that in comparison to leaves, the green fruits of *Nerium oleander* and *Rosa* sp. had higher Car/Chl ratio due to increased xanthophyll cycle components (violaxanthin, antheraxanthin and zeaxanthin) and a lower chlorophyll content. This resulted in a photoprotective xanthophyll cycle that is more functional under high light in green fruits than in leaves. This has also been reported for apple (Cheng and Ma, 2004) and grapevine (Young et al., 2016) and it is speculated that this exists in non-foliar photosynthetic organs to reflect a common strategy for photosynthetic green tissues under similar low oxygen conditions (Kyzeridou et al., 2015).

Some canopy manipulations, such as leaf removal in the fruiting zones are, however, utilized to increase light penetration to the berries (reviewed in Reynolds, 2010). A significant number of studies have investigated the impacts of leaf removal on berry development and ripening. Depending on the cultivar, the objectives range from improving the acid balance (Hunter and Visser, 1990; Toda et al., 2013; Baiano et al., 2015); improving anthocyanin/color stability (Chorti et al., 2010; Sternad Lemut et al., 2011; Lee and Skinkis, 2013; Baiano et al., 2015; Song et al., 2015; Guan et al., 2016; Yu et al., 2016; Pastore et al., 2017); increasing specific secondary metabolites such as volatile aroma precursors (Staff et al., 1997; Tardaguila et al., 2010; Feng et al., 2015; Song et al., 2015; Suklje et al., 2016; Young et al., 2016) or lowering of metabolites that are perceived negatively in the grapes/wines (Sala et al., 2004; reviewed in Sidhu et al., 2015). One of the main outcomes of leaf removal in the bunch zones is the accumulation of protective phenolic compounds i.e., anthocyanins (Lee and Skinkis, 2013; Guan et al., 2016; Lee, 2017) and flavonols (Yu et al., 2016; Pastore et al., 2017), as well as changes to volatile aroma compounds i.e., the norisoprenoid, β-damascenone (Feng et al., 2015; Young et al., 2016) and monoterpenes (Song et al., 2015; Young et al., 2016). These studies have all highlighted the adaptability of the grapevine berries to the changed microclimate and have also provided scope to investigate mechanisms of perceiving and adapting to the stresses linked to changes in microclimate.

Taking advantage of a validated experimental setting where light exposure (to the bunch zone) was the major environmental factor significantly altered by a classic leaf removal treatment in a model Sauvignon Blanc vineyard, the mechanism of berry acclimation to increased light exposure (Young et al., 2016) was targeted in this study. A pertinent result from the phenotyping and metabolite profiling was that none of the parameters and metabolites measured indicated a compromised primary growth/development and ripening of the berries under the increased exposure. Metabolically, the berries responded to increased light exposure by producing specific secondary metabolites that have photo-protective and/or antioxidant functions. The data generated in the targeted metabolite profiling of the berries lead to the conclusion that the berries mitigated the stress with metabolite reprogramming to acclimate to the increased exposure and that the response was strongly influenced by developmental stage. Although sugars, organic acids, chlorophylls and major photosynthetic pigments (β-carotene and lutein) were not affected by the increased light exposure; specific monoterpenes and photoprotective xanthophylls (zeaxanthin, antheraxanthin, and lutein epoxide) were shown to be increased (Young et al., 2016). These results raised an important question: How were primary metabolism and developmental patterns maintained, despite the light stress-response and metabolic reorganization activated in the exposed berries?

Our primary approach toward achieving these aims was to take a global transcriptional snapshot of gene expression at various berry developmental stages using RNA Sequencing (RNASeq) to thereby create an overview of the effects of elevated light exposure on berry development and ripening. Using this global overview, we were able to target specific metabolic pathways of which gene expression was most significantly affected by the treatment. We could further explore what affects these alterations in gene expression could have on accumulation of metabolites involved in these affected pathways to ultimately determine how berry growth and primary metabolism was maintained despite the activation of stress response mechanisms previously reported (Young et al., 2016).

## Materials and methods

### Experimental design, agronomical treatments, and sampling strategy

The *Vitis vinifera* cv. Sauvignon blanc grapes that were the research materials for this study were harvested from an experimental vineyard located in Elgin region of South Africa during the 2010/2011-harvest season. The complete details pertaining to the climatic measurements, vineyard layout, viticultural practices and sampling strategy of the relevant samples have been performed according to an established field-omics workflow (Alexandersson et al., 2014) and are available in Young et al. (2016). Briefly, grapes were sampled from twelve biological replicates (or panels with six panels per row; and six panels per treatment) in two adjacent vineyard rows (NW-SE row orientation). Each individual biological replicate (panel) consisted of four consecutive vines. The leaf-removal treatment included leaf and lateral shoot removal applied in the bunch zone on the SE-facing side of the canopy at EL29. This leaf-removal treatment was applied to every alternate panel creating a “checkerboard” plot layout where a control panel was always adjacent to an exposed panel (both within a row, and between rows) (Young et al., 2016).

The berries were sampled at green- (pea-sized) (EL31) (Eichhorn and Lorenz, 1977), pre-véraison- (EL33), véraison- (EL35), and the ripe-stage (EL38; corresponding to the commercial harvest date) from control (shaded) and exposed vine panels after which it was frozen in liquid nitrogen in the field. The seeds were removed from the frozen berries in the laboratory and the whole berries, including skins and pulp, were kept at −80°C until subsequent analyses were performed.

### Transcriptional analysis

#### RNA extraction and sequencing

Total RNA was extracted from three out of the six biological replicates sampled at four developmental stages under both exposed and control conditions according to an established protocol (Reid et al., 2006). Each of the 24 samples was subjected to DNAse1 treatment (Sigma-Aldrich, Saint-Louis, MO, USA) to eliminate contamination with genomic DNA. The concentration and purity of the extracted RNA samples were established using a Nanodrop 2000 Spectrophotometer (Thermo Scientific, Wilmington, DE, USA) and the integrity of the samples were confirmed through analysis of a Bioanalyzer Chip RNA 7500 series II (Agilent, Santa Clara, CA, USA) according to the manufacturer's instructions.

After achieving each of the quality control criteria, poly(A) mRNA was prepared for each of the RNA samples and sequenced through an Illumina HiSeq 1000 sequencer according to manufacturing protocols (Illumina Inc., San Diego, CA, USA). The reads generated from the sequencing procedure were aligned to the V1 version of the *V. vinifera* genome (PN40024) using version 2.0 of the TopHat software (Trapnell et al., 2012), allowing a maximum of two nucleotide mismatches. Cufflinks software (version 2.0) was subsequently used in order to assemble transcripts from generated sequence reads (Trapnell et al., 2012), hereby calculating the transcript abundance of each gene in the form of an FPKM value (expected fragments per kilobase of transcript per million fragments mapped). For the purpose of determining which transcripts show differential expression between treatments, CuffDiff (version 2.0) was used after transcript abundances were determined (Trapnell et al., 2012).

#### RNASeq expression data analysis

When comparing the entire transcriptomes of each of the samples included in this study, Pearson correlations were calculated using R (version 3.3.1) in RStudio (version 0.99.903) and the visualization of the results in the form of a correlation matrix were performed using Microsoft Excel (version 14.1.0).

Gene Ontology (GO) Enrichment analyses of the entire gene lists that showed non-significant differential expression between exposed and control samples at each phenological stage were performed in the BiNGO application in Cytoscape (version 3.4.0) using the Benjamini and Hochberg False Discovery Rate Correction metric. These genes will be further referred to as “unaffected.” GO terms were considered significant with a *p*-value smaller than 0.05.

In order to evaluate genes that were significantly affected by elevated light, the results generated from the differential expression analysis were implemented in a three step process according to different selection criteria. The first step was to perform GO enrichment analysis of all the genes that were significantly differentially expressed (*q* ≤ 0.05) under exposed conditions at each developmental stage in order to evaluate the effect that the treatment had on the berry transcriptome throughout development. Next, two distinct thresholds were chosen based upon the number of genes generated that would be most appropriate for subsequent analyses. The first threshold was set to include all differentially expressed genes with a log_2_ fold change greater than 1.5 and smaller than −1.5 when comparing the expression of exposed to control genes in order to generate a large list of highly significantly affected genes for the purpose of clustering analysis. This would allow for the identification and evaluation of the most prominent expression profiles of the genes affected by increased exposure without specifically focusing on individual genes. The second threshold was set to include only differentially expressed genes with a log_2_ fold change greater than 2 and smaller than −2 for the purpose of focusing on the individual genes that were most affected by increased exposure.

GO enrichment analysis of significantly enriched expression profile clusters of genes expressed at a log_2_ fold change (log_2_FC) greater than 1.5 between exposed and control grapes during at least one of the phenological stages were performed using the online analysis tool, AgriGO (Du et al., 2010) using the Fisher statistical method with the Yekutieli False Discovery Rate multitest adjustment metric. Significantly enriched GO terms (*p* < 0.05) were further visualized and summarized using the Reduce + Visualize Gene Ontology Web Server (http://revigo.irb.hr; Supek et al., 2011).

For the purpose of performing clustering analysis to infer which genes conform significantly to predetermined gene expression profiles, the Short Time-Course Expression Miner (STEM) was implemented (Ernst et al., 2006). Visualizations of the abovementioned differential expression analyses were performed using Microsoft Excel and Powerpoint (version 14.1.0).

The putative developmental biomarkers were identified and further explored in a three step process. Firstly, the molecular biomarkers of the control grapes representing the two most distinct developmental phases (i.e., green stages vs. ripening stages) were identified by implementing a previously established method (Zamboni et al., 2010). Putative biomarkers that represent the transcriptional difference between the green and the ripening grape berry stages were identified. A two-class OPLS-DA model was generated by representing the expression of green, control berry samples (EL31 and EL33) as its own class as a reference against expression of ripening, control berry samples (EL35 and EL38) set as the second class using SIMCA (version 14.0). An S-plot was subsequently generated to identify the loading correlation coefficient of each gene as described by Zamboni et al. (2010; Wiklund et al., 2008). The aim of this investigation was to generate a broad overview of the developmental progression of the grapes included in this study and therefore, a less stringent correlation cut-off was implemented than in previous studies to identify genes with a loading correlation coefficient higher than 0.8 (positive biomarkers) and lower than −0.8 (negative biomarkers). The expression of positive biomarkers were significantly higher in ripening berries compared to green berries, whereas negative biomarker expression was significantly lower in ripening berries compared to green berries (according to the nomenclature adopted by Zamboni et al., 2010).

Secondly, to establish whether these identified control grape berry developmental biomarkers were comparable to those already established for grape developmental progression, molecular biomarkers identified in this investigation were compared to those published from two previous investigations. The first set of biomarkers included in this comparison was published by Zamboni et al. (2010) in which transcriptional elements unique to early berry development (EL33 and EL35) and late berry development (EL36 and EL38) were identified and named Class a and Class b genes, respectively. These biomarkers will be referred to as early and late developmental markers in subsequent sections of this publication. The second set of genes used to compare the development of the grapes included in this study was published by Palumbo et al. (2014) in which they identified so-called “switch genes” that are considered to characterize the unique transcriptional switch that occurs when grape berries transition from being green, photosynthesizing organs to becoming ripening, sink organs. This aforementioned study utilized transcriptional data generated from five red Italian grape cultivars as well as data generated from the grapevine transcription atlas (Fasoli et al., 2012). A Venn diagram was constructed using the Bioinformatics and Evolutionary Genomics platform (http://bioinformatics.psb.ugent.be/webtools/Venn/) by comparing the genes from the abovementioned studies and the molecular biomarkers identified in this study.

Finally, using the identified developmental biomarkers, the effect of the treatment on the progression of berry development was further explored. This was achieved by determining which of the identified biomarkers shared between this and previous studies were significantly affected by the leaf-removal treatment (and increased exposure) by evaluating the differential expression of these genes.

In order to determine how photosynthesis is affected on a transcriptional level by elevated light exposure, the appropriate gene accessions encoding proteins of PSI and PSII of the thylakoid membranes were obtained from the KEGG Pathway database for *V. vinifera* (http://www.kegg.jp/kegg-bin/highlight_pathway?scale=1.0&map=vvi00195&keyword).

#### Quantitative real-time polymerase chain reaction (RT-PCR)

In order to validate the accuracy of the gene expression patterns observed in the results generated through RNASeq analysis, RT-PCR was performed using the Applied Biosystems 7500 Real-time PCR System. For these verification assays, total RNA was extracted from three of the six biological replicates originally harvested for metabolic and RNA Seq analyses using the Spectrum™ Plant Total RNA Kit (Sigma-Aldrich, Saint-Louis, MO, USA). cDNA was synthesized from the total RNA using the SensiFAST™ cDNA Synthesis Kit (Bioline, London, UK) and RT-PCR was performed using the KAPA SYBR®FAST qRT-PCR Kit according to the manufacturer's instructions (Kapa Biosystems, Cape Town, South Africa). Six genes were selected as targets for the PCR reactions based on their expression patterns in response to the treatment as reported by the RNASeq analysis. Four of these target genes were upregulated in response to the treatment by a log_2_FC greater than 2 at various developmental stages (VIT_10s0116g00410, VIT_18s0001g03470, VIT_05s0020g04110, VIT_02s0025g04060). The other two of the target genes were related to photosynthesis and were significantly upregulated by elevated light exposure in the green berries (VIT_01s0010g03620, VIT_19s0014g00160). Appropriate primers were designed using QuantPrime (Arvidsson et al., 2008). These primers, their sequences and their characteristics are summarized in Table S1. All PCR reactions were performed in triplicate. The normalization and absolute quantification of the expression levels of each of the six genes were performed using the Linear Regression Efficiency (LRE) method using LRE Analyzer software (Rutledge and Stewart, 2008; Rutledge, 2011).

### Metabolite analysis

Extractions and subsequent metabolite analyses were performed from three out of the six available biological repeats that represented the biological triplicates sampled at four developmental stages under both exposed and control conditions.

#### Amino acid analysis

The extraction and HPLC analysis of amino acids in berry samples was performed as described in Antalick et al. (2010), with minor changes. Frozen homogenized berry tissue (200 ± 10 mg) was weighed into 2 mL microfuge tubes and 0.5 mL of 70% (v/v) methanol [containing 25 mg/L of each of the two internal standards (IS), sarcosine and norvaline] was added. Samples were briefly vortexed and sonicated for 10 min at room temperature. After sonication, the samples were centrifuged at 1,250 rpm for 5 min and 200 μL of the supernatant was transferred to amber vials, crimp-sealed and if not analyzed immediately stored at −4°C. Each biological replicate was extracted and analyzed in triplicate. The extracted amino acids were derivatized before analysis on HPLC as described in Suklje et al. (2016).

Major amino acids (AAs) were identified based on their retention times with respect to authentic standard elution and quantified using external standard calibration based on standard curves plotted using the peak areas vs. the standard concentrations. Concentrations were normalized to the IS amount and the sample fresh weight (FW) to obtain the AA concentrations per fresh berry weight (mg/g FW).

#### Quantification of phenolic compound contents

All authentic standards namely quercetin-glucoside; catechin, epicatechin as well as caftaric acid and caffeic acid as well as the HPLC grade solvents used for sample extraction and separation such as methanol (MeOH, 99.0%), acetonitrile (99.0%), hydrochloric acid (HCl), and the orthophosphoric acid (H_2_PO_4_, 99.0%) were acquired from Sigma Aldrich (Steinheim, Germany).

Homogenized grapevine berries (200 ± 10 mg) were weighed and 0.5 ml of acidified MeOH (70%; adjusted to pH 1.5 with HCl) was added to each vial, which was then vortexed and sonicated for 15 min at room temperature. After sonication, the samples were centrifuged at 1400 rpm for 5 min and 200 μL of the supernatant was collected and added into amber vials, crimp-sealed for HPLC analysis. Extraction was done in triplicate, in a dark room away from direct light. Extracted flavonoids and phenolic acids in berries were separated and quantified using an Agilent 1100 series HPLC system (Agilent Technologies©, Palo Alto, California, USA) equipped with a diode array detector (DAD) and controlled by a ChemStation Rev. A.10.02 software (Agilent Technologies©). The column used was a Phenomenex Prodigy ODS-2 (4.6 × 150 mm, 5 μm) preloaded with Phenomenex Prodigy guard cartridge (2.1 mm × 100 mm, 1.7 um). The mobile phases were composed of 15% (v/v) H_2_PO_4_ (A) and 80% acetonitrile containing 20% A (B) and the flow rate was 1 mL/min. The gradient elution conditions started with a linear gradient from 6 to 31% B for 68 min following with another linear gradient from 31 to 65% B for 5 min. Then, the gradient was kept constant at 65% B for 5 min and was decreased from 65%, back to the starting conditions at 6% B for 5 min. The system was re-equilibrated at 6% B for another 10 min before the next injection. The injection volume was set at 20 μL and the column temperature at 40°C.

The major flavonoids and phenolic acids in grapevine berry samples were identified based on their retention times with respect to authentic standard elution and quantified using external standard calibration based on standard curves plotted using the peak areas vs. the standard concentrations. These chromatographic peaks were obtained using the following DAD wavelengths: 280 nm for flavan-3-ols; 360 nm for flavonols and 320 nm for the phenolic acids. Compounds without available standards were quantified using the calibration parameters from quercetin-glucoside (all flavonols) and caftaric acid. The concentrations in samples were normalized to the sample fresh weight (FW) to obtain the sample amount per berry FW (μg/gFW). Table S2 summarizes the retention time and calibration parameters of all standards used in this analysis.

#### Lipophilic-oxygen radical absorbance capacity (L-ORAC) assay

L-ORAC analysis was performed by the Antioxidant Research Unit (Cape Peninsula University of Technology, South Africa) on three biological replicates (in triplicate) harvested at EL33 and EL38, respectively.

#### Statistical analysis

The concentrations generated from the analysis of amino acids and phenolic compounds of the grapes were subjected to multivariate data analysis using Statistica (version 13.0). A repeated measures analysis of variance (ANOVA) was performed to identify the relationship between the increased exposure treatment and the concentrations of the measured compounds (AAs and Phenolic compounds). A Fisher LSD *Post-Hoc* test was conducted for each compound to confirm whether the concentration of the compound was statistically significantly affected by the treatment (*q*-value).

Basic statistical analysis of data generated from the L-ORAC assay was conducted in Microsoft Excel (version 14.1.0) using a paired *t*-test to determine whether exposed grapes had significantly higher lipophilic antioxidant capacity than control grapes at EL33 and EL38.

## Results

### Overview of the transcriptional data generated

In this study, RNASeq was performed with 24 Sauvignon blanc berry samples representing grapes from shaded (control) and exposed (treatment) microclimates at four developmental stages from a highly characterized vineyard. A summary of the parsed reads from each of the samples and the number of reads that mapped onto the *V. vinifera* cv. Pinot noir reference genome (PN40024) are included in Table S3. The complete RNASeq dataset is available in the NCBI's GEO under the series accession, GSE98873.

In order to compare the complete transcriptomes generated for the 24 grape samples, a correlation matrix was generated by implementing a Pearson's correlation coefficient as a distance metric (Figure S1). The resulting matrix revealed that one sample harvested at EL38 did not correlate strongly to the rest of the EL38 samples, but rather to samples taken at EL33. Not only were the other 23 samples closely grouped according to their specific developmental stage, targeted metabolite profiling of the same grape samples previously confirmed the close grouping of all the EL38 samples (Figures 3, 4 in Young et al., 2016). This sample was treated as an outlier (anomaly) and excluded from all subsequent analyses.

**Figure 1:**
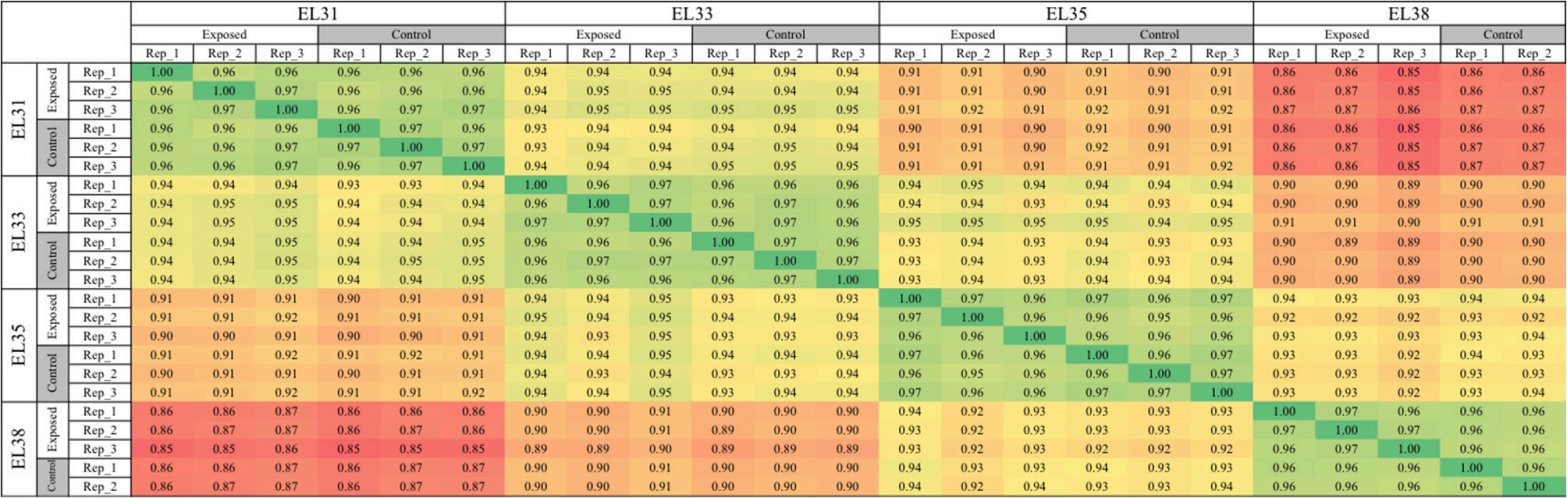
Pearson correlation matrix of the entire transcriptomes of 23 samples representing exposed and control grapes at four developmental stages (EL31, EL33, EL35, and EL38).

The Pearson correlation matrix was reconstructed including only the 23 remaining samples and is presented in Figure 1; the matrix shows a strong correlation between grapes from the same developmental stage, regardless of the viticultural treatment implemented. Furthermore, gene expression of green berries was more closely correlated between EL31 and EL33 stages than with the two consecutive ripening stages, EL35 and EL38. The correlation matrix also provided confidence in the experimental design and sampling strategy since the biological replicates of the control and exposed treatments confirmed the repeatability of the effect that the leaf removal treatment had on the berry transcriptome at each developmental stage.

Out of the 29,970 genes represented in this version (V1) of the grapevine genome, the expression of 5,050 genes (16.5%) could not be detected across any of the observed developmental stages and treatments and the enriched GO terms representing these genes are summarized as Figure S2 (as represented by Revigo). A further 4,715 genes with FPKM expression values lower than the recommended reliable RNASeq threshold of an FPKM = 1 (Warden et al., 2013; Massonnet, 2015) throughout all developmental stages and treatments were excluded from further analyses.

RT-PCR analysis of six genes that showed significant upregulation in response to the exposure treatment was conducted and validated the accuracy of the RNASeq results (Figure S3). Predominantly, the general expression trend throughout development of each of the genes was similar when comparing the RNASeq and RT-PCR results for control and exposed grapes. These initial analyses not only established confidence in the experimental design and the repeatability among biological replicates, but it further established the accuracy of the RNASeq method and subsequent results generated.

### Developmental biomarker analysis

In total, the expression of 4,975 genes was identified as developmental phase-specific biomarkers responsible for the greatest transcriptional differences between the green and ripening developmental stages. 2,242 and 2,733 of these genes were positively and negatively correlated (Correlation value ≥ 0.8) to the separation, respectively (Table S4).

The expression of these markers was comparable to previously established markers for grape berry development (Zamboni et al., 2010; Palumbo et al., 2014). Furthermore, the expression of 81% of these shared markers developmental markers were not affected by the treatment. The remaining nine genes responsible for the 19% of developmental biomarkers that were affected by the treatment included an auxin-responsive gene (SAUR29; VIT_16s0098g01150), two genes encoding protein subunits of photosystem I and II (VIT_12s0028g01080, VIT_05s0020g03180) and a calmodulin-binding heat shock protein (VIT_14s0006g01030). The results are summarized in Figure S3.

### Transcriptional response of the berries to increased exposure

#### Transcripts that were unaffected

**Figure 2:**
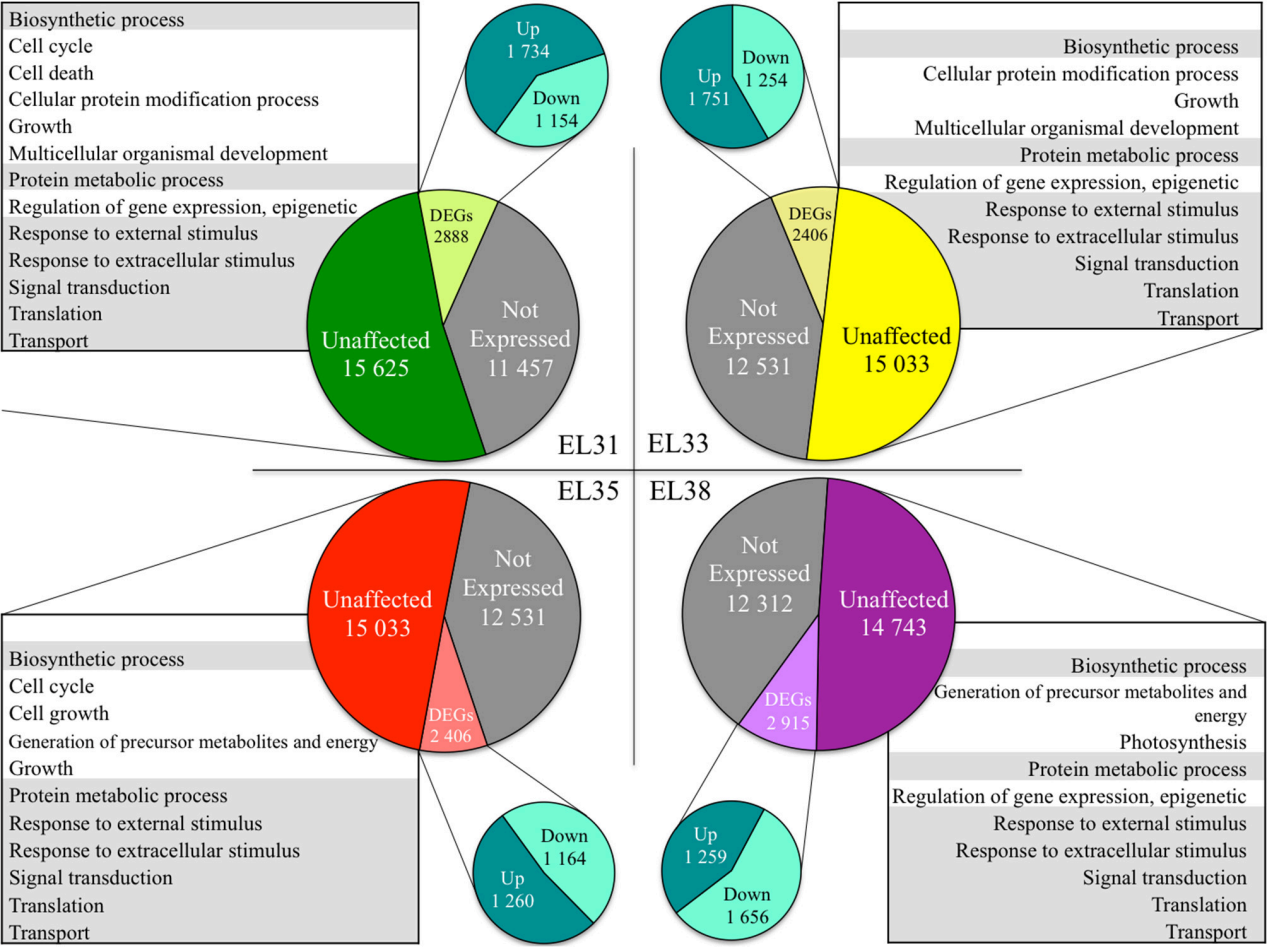
Pie charts representing the number of genes in the grapevine genome either not expressed, significantly unaffected (*q* ≥ 0.05) and significantly differentially expressed in response to elevated light (*q* ≤ 0.05) at the four phenological stages, respectively. The GO terms significantly enriched representing the genes unaffected by the treatment at each phenological stage are summarized in tables. Gray shading represents GO terms that were commonly unaffected throughout berry development.

The number of annotated genes that were either not expressed, unaffected by the leaf removal treatment or differentially expressed when comparing exposed to control grapes at each of the phenological stages are summarized in Figure 2.

GO enrichment analysis of the genes statistically unaffected by the light treatment revealed that GO terms associated with growth and development were enriched throughout development. Among these were GO terms related to “Biosynthetic process,” “Signal transduction,” “Protein metabolic process,” “Translation,” “Transport,” and “Response to external stimulus” (Figure 2). Furthermore, during the developmental stages in which the berries were photosynthetically active and growing in size (EL31, EL33, and EL35), genes associated with the GO terms “Growth” and “Multicellular organismal development” were unaffected by the treatment at EL31 and EL33 as well.

#### Transcripts that were differentially expressed as a consequence of the treatment

**Figure 3:**
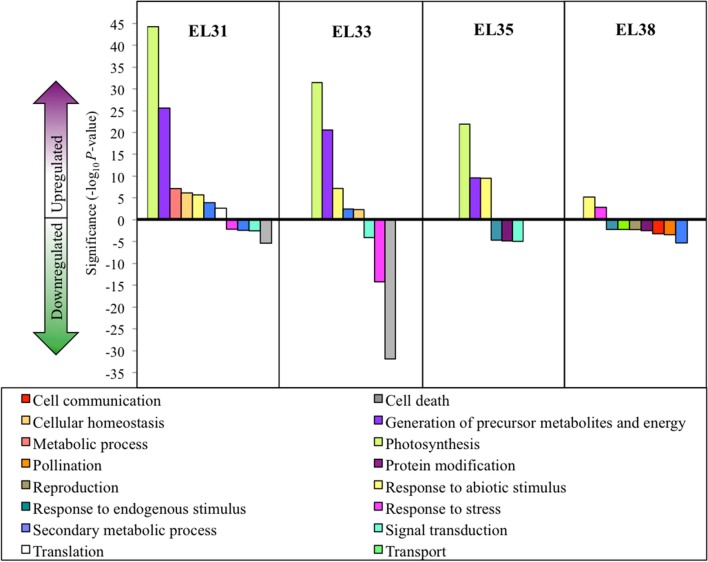
Grape berry transcripts that are significantly differentially expressed in response to elevated light exposure at four phenological stages. Significantly enriched GO categories (*q* ≤ 0.05) at each phenological stage. Significance is represented as log_10_ *P*-values of each GO category with positive values indicating upregulation and negative values indicating downregulation.

By implementing Cuffdiff software, transcripts that were significantly differentially expressed (*q* ≤ 0.05) when comparing exposed to control grapes could be identified. For each of the four developmental stages being evaluated, the percentage of differentially expressed genes (DEGs) were calculated and the genes that were either significantly up or downregulated in response to the leaf removal treatment could be explored by implementing GO enrichment analysis. The results of these analyses are summarized in Figure 3.

These results revealed that grape berries were most significantly affected by the treatment on a transcriptional level during the early developmental stages (EL31 and EL33) and the global description of the biological processes these gene groups were involved with, shared a high degree of similarity between EL31 and EL33 grapes. GO terms associated with photosynthesis and the generation of precursor metabolites and energy were very highly upregulated in exposed grapes until véraison. In the green grapes, especially during EL33, genes associated with the GO terms “cell death” and “response to stress” were among the most significantly downregulated functional groups, exclusively representing genes associated with disease and nematode resistance.

Although ripe berries had the highest number of DEGs in response to the treatment, the enrichment of the GO terms affected by the treatment were lower in comparison to the preceding developmental stages. These enriched GO terms were further associated with genes that were significantly downregulated in response to the treatment as opposed to the preceding stages that were dominated by upregulation in response to increased exposure.

**Figure 4:**
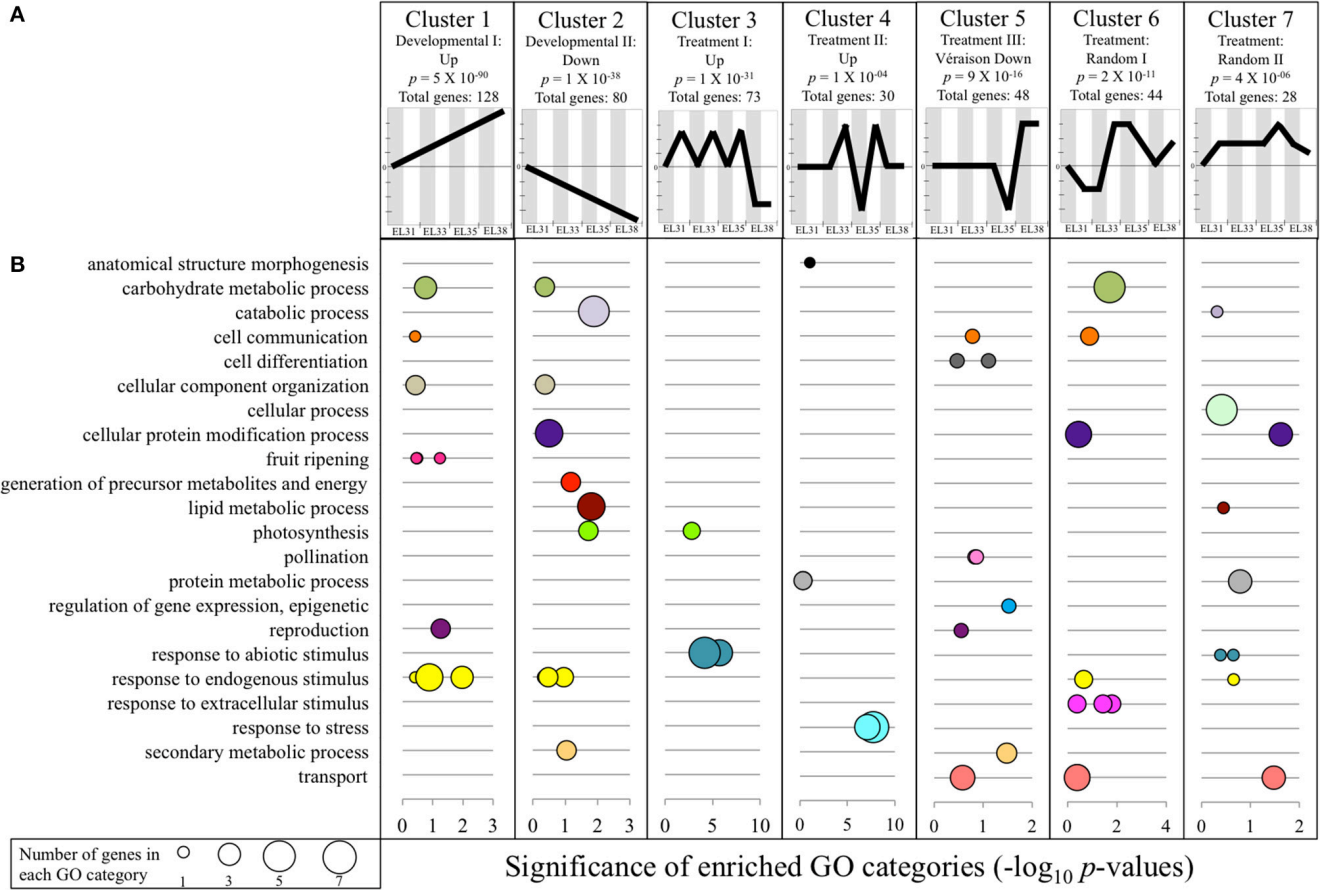
Expression clustering analysis results of all genes differentially regulated (*q* ≤ 0.05) by a log_2_ fold-change higher by a factor of 1.5 (1.5 ≤ log_2_FC ≤ −1.5) in response to elevated light exposure during at least one of the four phenological stages. **(A)** The seven expression profiles to which a significant amount of genes aligned. Shaded columns (gray) indicate the expression of the genes in that specific cluster under control conditions and white columns indicate expression of the same genes under exposed conditions at the developmental stage indicated in the X-axis below. Significance is indicated in each cluster profile representation in the form of a *P*-value. **(B)** Functional GO subcategories of each significantly enriched expression cluster summarized within representative GO terms as summarized by ReviGO. Significance is represented as −log_10_ *P*-values of each subcategory; the size of each data circle indicates the number of genes that is represented within each enriched GO term.

Out of the 29970 genes included in the grapevine genome, 723 genes showed either significant up or downregulation with a factor greater than 1.5 (log_2_FC) during at least one developmental stage in response to the elevated light treatment. Clustering analysis revealed that the expression of 431 of these genes could be grouped to seven expression profile clusters as predetermined by the STEM software (Figure 4A), with the GO subcategories provided in Figure 4B and the genes within each cluster summarized in Table S6.

Two of these identified expression clusters (clusters 1 and 2) were represented by genes that followed the predicted developmental progression whilst simultaneously being affected by the treatment. Cluster 1 (*p* = 5E^−90^; total of 128 genes) represented genes that were simultaneously driven by the increased exposure treatment as well as developmental cues. Several of the functional annotations were associated with the progression of grape berry development, but also secondary metabolic processes linked to abiotic stress responses. Examples of genes within cluster 1 included three Ethylene-responsive transcription factors (VIT_07S0031G01980, VIT_01S0150G00120, VIT_14S0108G00050), a 2-oxoglutarate/malate carrier protein (UCP5; VIT_18S0001G07320) that has been proposed to be involved with acid regulation in grape berries (Chen et al., 2015), a Galactinol synthase (GolS4; VIT_01S0127G00470) involved in the synthesis of the osmoprotectant oligosaccharide, raffinose, a gene encoding a Gamma-aminobutrytic acid transporter (VIT_13S0074G00570), two genes encoding enzymes involved in the phenylpropanoid/flavonoid pathway (anthocyanidin 3-O-glucosyltransferase, VIT_12S0034G00130; Flavanone 3-hydroxylase, VIT_16S0098G00860), as well as the early light-inducible protein (ELIP1, VIT_05S0020G04110) involved in the inhibition of chlorophyll biosynthesis.

Interestingly, 64 of the genes represented by cluster 1 were also identified as developmental biomarkers (Figure S4) of which five were shared with the analyses of Zamboni et al. (2010) and Palumbo et al. (2014). One of these genes is a 9-cis-epoxycarotenoid dioxygenase encoding gene (NCED; VIT_02S0087G00930) responsible for the degradation of carotenoids synthesized during the early developmental phases to produce the plant hormone, abscisic acid (ABA) that further plays a pivotal role in plant adaptation to stress.

Cluster 2 represented 80 genes that showed significant downregulation throughout development under exposed conditions, while simultaneously following the same developmental progression. Among the GO terms associated with this cluster were “lipid metabolic process” that represented two senescence-associated genes (SAG101, VIT_14S0066G01830, VIT_14S0066G01820) involved in stress-related signaling, as well as the GO terms “photosynthesis” and “generation of precursor metabolites and energy” that both represented genes that encode a photosystem II PsbO protein (VIT_18S0001G11710), an LHB1B1 light harvesting protein (VIT_12S0028G00320) and another a polyphenol oxidase chloroplast precursor (VIT_10S0116G00560). Cluster 2 also contained an Alanine-glyoxylate aminotransferase encoding gene (Alanine-glyoxylate aminotransferase 2 3, mitochondrial, VIT_08S0058G00930) that plays a central role in the photorespiratory pathway and a gene encoding a trehalose-6-phosphate phosphatase (VIT_00S0304G00080) that is known to have an indispensible role in normal plant growth and development. Furthermore, 18 of the genes represented by cluster 2 have been identified as negative biomarkers in this study (Figure S4).

Clusters 3 and 4 (Figure 4) contained genes that were highly responsive to the elevated light exposure treatment regardless of the developmental profile. The genes represented by these clusters show strong functional associations to the activation of several protection mechanisms of the photosynthetic machinery, activated at either the first (EL31) or the second green developmental stage (EL33). Several heat shock protein (HSP) encoding genes, including the well-known abiotic stress signaling regulator, heat shock factor 2A (VIT_04S0008G01110), alongside its putative co-activator, Multiprotein-bridging factor 1 (VIT_11S0016G04080), as well as small HSPs formed part of these clusters.

Cluster 3 further represented several genes that contributed to the GO term, “photosynthesis.” These included a gene encoding a chloroplastic carbonic anhydrase (VIT_14S0066G01210) critical in the maintenance of the rate of photosynthetic CO_2_ fixation, and a photosystem II protein encoding gene (PsbP, VIT_13S0019G00320) that forms part of the oxygen evolving complex of PSII, specifically contributing toward its stabilization. Furthermore, a WUSCHEL encoding gene (VIT_18S0001G10160) was present in this cluster that represents a member of a transcription factor gene family involved in reproductive organ development, hormone signaling and abiotic stress response in several plant species.

The 30 genes represented by expression cluster 4 show significantly higher expression from EL33 until véraison after which the expression of these genes was unaffected in ripe berries in response to the treatment. Among the 24 genes within this cluster that had been functionally annotated, an FtsH protease encoding gene (VIT_14S0108G00590), known to be involved in the efficient turnover of the D1 protein of PSII in response to photooxidation, as well as a Calmodulin encoding gene (VIT_18S0122G00180) known to be involved in stress perception and signaling related to cellular calcium ion (Ca^2+^) concentration in plants were included. Furthermore, this cluster represented genes encoding a galactinol synthase (VIT_07S0005G01970), a Methyl jasmonate esterase (VIT_00S0253G00150) and a 2-oxoglutarate-dependent dioxygenase (VIT_05S0049G00220) among others. Clusters 3 and 4 therefore point toward the activation and maintenance of light stress mitigation strategies during the green developmental stages.

The remaining three clusters (clusters 5, 6, and 7) represented genes that were differentially affected by elevated light exposure according to neither a unique developmental pattern nor consistently by the treatment (Figure 4; Table S6). Due to the random and complex nature of their transcriptional responses, these gene clusters were not further investigated for the purpose of this study.

**Figure 5:**
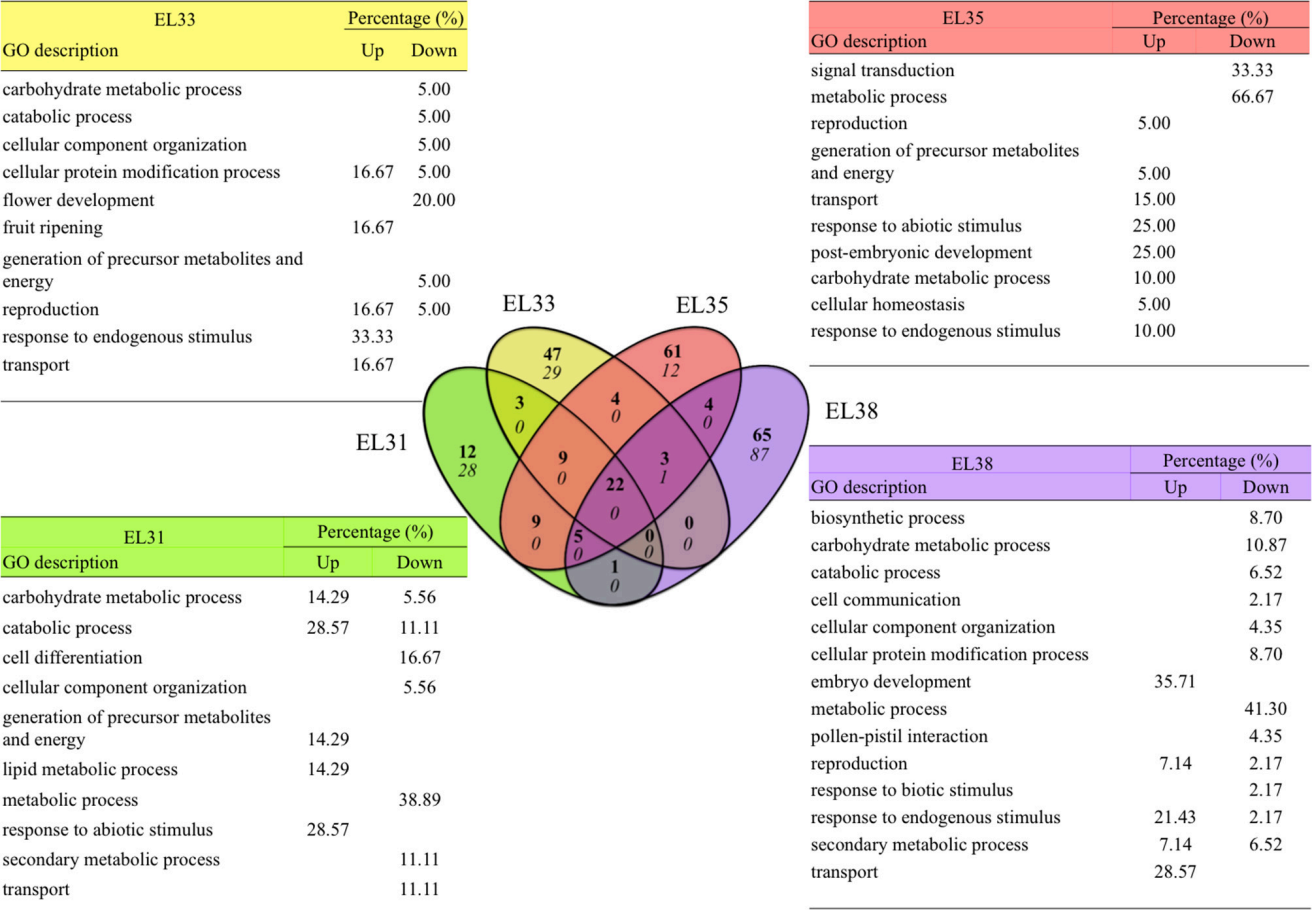
Venn diagram summarizing the functional associations of the genes up or downregulated by a Log_2_ fold-change greater than 2 and smaller than −2 when comparing exposed to control grapes at four phenological stages. The number of upregulated genes is indicated in bold and the number of downregulated genes is italicized in the Venn diagram and GO annotations of the genes uniquely highly differentially regulated at each developmental stage. GO descriptions indicate GO terms that were representative of each gene group, percentages indicate the percentage of genes that are represented by each GO description.

In the second step taken to elucidate which transcriptional elements are the most significantly affected by elevated light exposure at each individual stage, genes that show a Log_2_ fold change (Log_2_FC) either higher than 2 or lower than −2 in exposed compared to control grapes were further explored. In total, 245 and 157 genes were up and downregulated in exposed compared to control grapes according to these criteria, respectively. These genes are listed in Table S5 and their functional associations are summarized in Figure 5.

Among these 245 significantly upregulated genes, 185 were uniquely upregulated at very high levels at each developmental stage investigated with 12, 47, 61, and 65 genes upregulated (Log_2_FC ≥ 2) at EL31, EL33, EL35, and EL38, respectively. Out of the 157 genes that were most significantly downregulated (Log_2_FC ≤ −2), 156 of these genes were uniquely downregulated at either EL31 (28 genes), EL33 (29 genes), EL35 (12), or EL38 (87) in response to elevated light exposure. Several genes were similarly upregulated in various developmental stages (Figure 5). These genes, their functional annotations and the significance of their differential expression (*q*-values) are summarized in Table S7.

### Metabolic processes most affected by elevated light exposure

The global transcriptional analysis of Sauvignon blanc grape berries yielded insights into which metabolic processes are most affected by elevated light exposure. Gene expression involved in photosynthesis and the synthesis of flavonoid compounds were most significantly activated by the light treatment, which warranted further investigation into how subsequent primary and secondary metabolism of the grape berries was affected by the treatment. In order to investigate these metabolic processes, the synthesis and degradation of the amino acid transcription and composition was further investigated and explored in the context of how this AA metabolism may affect secondary metabolism in response to elevated light exposure in the berry bunch zone.

#### Protection of the photosynthetic machinery

**Figure 6:**
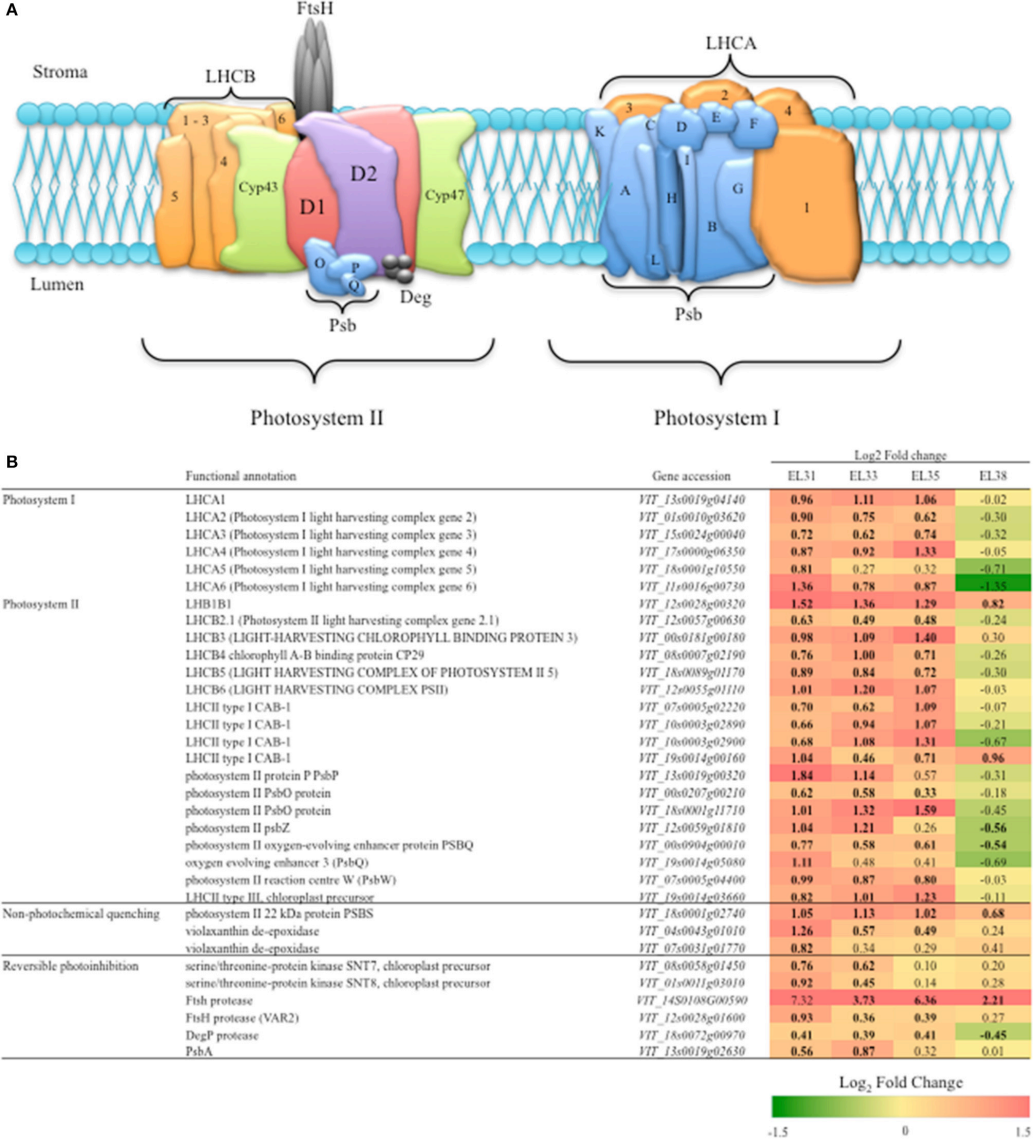
Grape berry photosynthesis and mechanisms of photoprotection. **(A)** A Simplified schematic model of the photosynthetic machinery located in the thylakoid membrane within the chloroplasts. **(B)** A table of representative candidate genes involved in photosystem I and II and two mechanisms of photoprotection in the form of non-photochemical quenching and reversible photoinhibition (RPI), their accessions and the log_2_ fold-change when comparing their expression levels (FPKM) between exposed and control grapes at each developmental stage. Significant differences in expression between exposed and control grapes are indicated in bold.

The 24 genes included in the investigation of PSI and PSII, their functional annotations and the Log_2_ fold change of the expression of each gene when comparing exposed to control grapes at each developmental stage is summarized in Figure 6. Every gene included in this analysis was significantly upregulated (*q* ≤ 0.05) in response to the leaf removal treatment at EL31.

Similarly, during EL33 and EL35, most of the genes of PSI and PSII remained significantly upregulated with the exception of one LHCA gene (LHCA5, VIT_18s0001g10550), two Psb encoding genes (PsbP, VIT_13s0019g00320; PsbZ, VIT_12s0059g01810) that were unaffected from véraison onwards and a PsbQ (VIT_19s0014g05080) that was unaffected by elevated light exposure from EL33 onwards. Thereafter, at EL38, with the exception of one CAB encoding gene (LHCII type I CAB-1, VIT_19s0014g00160), all of the genes evaluated became either unaffected by the treatment or significantly downregulated in response to the treatment.

The genes putatively encoding enzymes involved in photoprotection mechanisms in grapevine have been acquired from *Arabidopsis* orthologs and the log_2_FC of their expression when comparing exposed to control grapes at each developmental stage and is also summarized in Figure 6. At EL31, all the genes encoding the enzymes of both non-photochemical quenching (NPQ) and reversible photoinhibition were significantly upregulated with exception of one FtsH protease-encoding gene (VIT_14S0108G00590). Although the abovementioned FtsH protease appeared to be highly upregulated (Log_2_FC = 7.32), it's expression proved to be highly variable among the biological replicates in this study and was therefore not significantly different when comparing exposed to control berries at EL31. The genes encoding NPQ associated proteins that include PsbS (VIT_18s0001g02740) and one violaxanthin deepoxidase enzyme (VDE) encoding gene (VIT_04s0043g01010) were strongly upregulated by the treatment at EL31. At EL33, however, the FtsH protease-encoding gene (VIT_14S0108G00590), putatively responsible for the degradation of damaged copies of the D1 protein, was most significantly and highly upregulated until the berries were ripe (EL38).

#### The effect of elevated light exposure on amino acid metabolism of developing grape berries

HPLC analysis was performed that yielded the concentrations of 23 amino acids at the four developmental stages. The amino acid (AA) concentrations generated for each of the samples generated are included in Table S8. The log_2_FC values and statistical significance between exposed and control grapes are summarized in Table 1. Among these 23 amino acids, the concentrations of eight of these were not affected by the leaf removal treatment at any of the developmental stages evaluated. The only amino acid that was affected by the leaf removal treatment throughout the entire berry development was Gly that was present at significantly higher concentrations from EL31 until EL38.

**Table 1:** The fold change (Log_2_) of the amino acid concentrations (mg/gFW) of developing grapes when comparing exposed to control berries at four phenological stages.

	**Exposed vs. Control (Log_2_ fold change)**			
	**EL31**	**EL33**	**EL35**	**EL38**
Ala	0.26	0.17	−0.29	0.02
Arg	−0.78	0.04	−0.50	−0.38
Asn	0.00	−0.99	−1.04	−1.08
Asp	−1.12	−0.53	−0.87	−1.05
Cys	0.00	−0.28	−1.06	−0.04
Cys-Cys	0.59	−0.06	0.01	0.39
GABA	0.26	0.14	0.39	0.63
Gln	−0.28	−0.82	−0.81	−0.80
Glu	−0.10	−0.14	−0.77	−0.01
Gly	1.55	1.15	0.98	0.31
His	−0.50	−0.11	−0.18	−0.19
Ile	0.00	2.13	−0.37	0.01
Leu	−0.77	0.16	−0.23	0.10
Lys	0.00	1.64	0.40	−0.03
Met	0.45	−0.28	−0.87	0.30
Orn	1.21	0.39	0.48	−0.02
Phe	−0.97	0.15	−0.68	−0.41
Pro	0.66	0.44	0.53	0.30
Ser	0.31	−0.26	−0.07	−0.20
Thr	−0.32	0.12	−0.02	−0.10
Trp	−0.20	−0.39	−0.64	−0.99
Tyr	−0.39	0.13	−0.26	0.01
Val	−0.18	0.56	−0.21	0.22
Tot AA	−0.23	−0.48	−0.56	−0.22

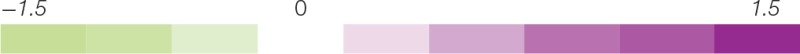

*Values that are statistically different between exposed and control grapes (q ≤ 0.05) are colored according to either higher or lower concentrations. These colors indicate higher or lower log_2_ fold changes between exposed and control grapes based on their concentrations but are not indicative of higher or lower concentrations themselves*.

Taken together these results revealed that, with the exception of Gly, most of the AA concentrations remained unaffected by the treatment until the onset of ripening, followed by the accumulation of significantly altered AA concentrations when comparing exposed to control grapes.

At véraison (EL35) 10 out of the 23 AAs measured were present at significantly lower concentrations in exposed grapes, including the four key nitrogen assimilation AAs, Asp, Asn, Glu, Gln, as well as Ala, Arg, Cys, Met and two aromatic AAs, Phe, and Trp. When the berries achieved ripeness at EL38, GABA, Met, Pro, and Val were present at significantly higher concentrations along with Gly whereas Arg, Asp, Phe, and Trp remained present at lower concentrations in exposed grapes. At this stage, His concentrations were also significantly lower when comparing exposed to control grapes.

**Figure 7:**
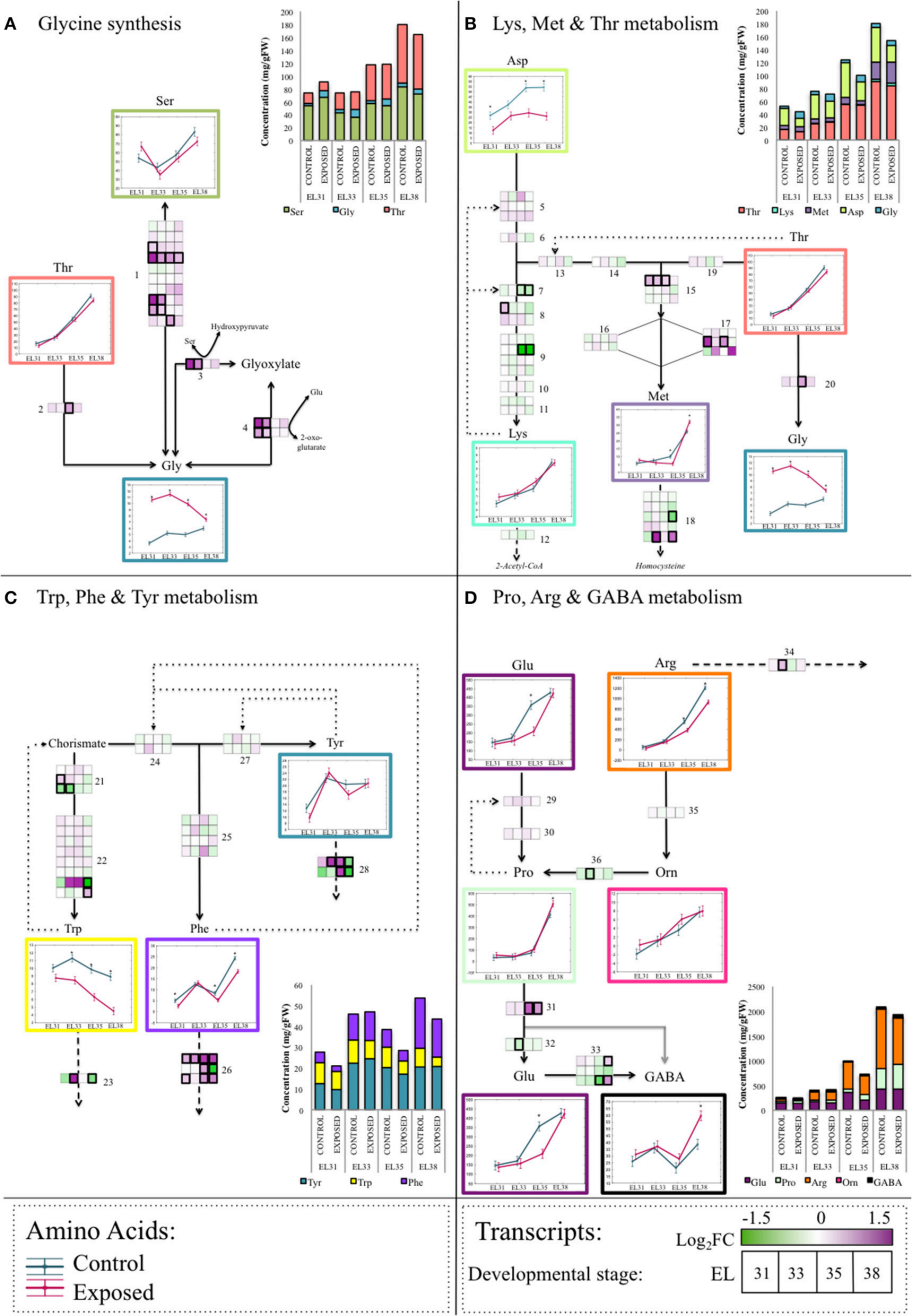
A summarized schematic representation of the four amino acid metabolite and transcriptomic networks analyzed in this study. Enzymatic steps are indicated as black arrowed lines, spontaneous (non-enzymatic) metabolic processes are indicated by gray arrowed lines. **(A)** The network representing the various pathways involved in Gly synthesis. **(B)** The superpathway of Lys, Met, and Thr synthesis from Asp. **(C)** The superpathway of Trp, Phe and Tyr synthesis from chorismate. **(D)** The superpathway of Pro, Arg, and GABA metabolism. Dotted lines represent feedback inhibition loops, whereas striped lines represent catabolic pathways of amino acids not included in this diagram. Blocks indicate the mean-centered log_2_ fold change of the FPKM expression value of the specific transcript encoding the particular enzymatic step at each berry developmental stage when comparing exposed to control samples. Significant differences between FPKM expression values between exposed and control grapes at a particular developmental stage is indicated by a bold frame around the specific gene. Amino acid concentrations [mg/g fresh weight (FW)] are represented as ANOVA line-plots where significant differences (*q* ≤ 0.05) between exposed and control grapes are indicated by an asterisks (^*^). Line graphs representing exposed and control samples are staggered along the x-axis representing the respective developmental stages. The genes represented by numbers are listed in Table S9.

To explore the transcriptional regulation of the synthesis and degradation of several of the AAs that were present at altered concentrations in response to the leaf removal treatment, four metabolic pathways including several of the altered AAs were targeted for further investigation. These metabolic pathways included Gly metabolism (Figure 7A), the superpathway of Lys, Met and Thr metabolism (Figure 7B), the superpathway of Trp, Phe, and Tyr metabolism (Figure 7C) and the pathway that involved Pro, Arg, and GABA metabolism (Figure 7D). The genes putatively involved in these metabolic pathways according to the current available gene annotation collection are indicated by numbers in the appropriate diagrams and are summarized in Table S9.

By evaluating these four AA metabolic pathways it became clear that transcription of the biosynthetic enzyme encoding genes were only marginally affected by the leaf removal treatment, whereas the genes encoding enzymes responsible for the degradation of many of the evaluated AAs were transcriptionally far more reactive to the treatment in the ripening period. The pathway depicting Trp, Phe, and Tyr metabolism (Figure 7C) is one example of this upregulation of AA catabolic enzyme encoding genes where the genes responsible for the synthesis of Phe and Tyr were not significantly affected by elevated light exposure at any of the berry developmental stages. The Phe ammonia lyase (PAL) encoding genes (VIT_06s0004g02620, VIT_08s0040g01710, VIT_13s0019g04460) and the Tyr aminotransferase encoding genes (VIT_00s0225g00230, VIT_00s0394g00040) respectively responsible for the degradation of Phe and Tyr were, however, significantly differentially expressed in response to the increased exposure at various stages of berry development.

Increased exposure had distinctly different consequences on grape AA metabolism when comparing green to ripening berries. An example of this developmental, stage-specific metabolism was evident in the upregulation of AA catabolic enzymes in the pathways involved in Gly synthesis (Figure 7A) whereby Gly synthesis from the catabolism of both Ser and Glyoxylate were higher in exposed grapes during the green berry stages under elevated light conditions. Conversely, during the berry ripening stages, the synthesis of Gly from the degradation of Ser and Thr by the upregulation of catabolic enzyme encoding genes were higher in exposed grapes. The degradation of several of these AAs will make their constituents, whether secondary compounds or other AAs, available as substrates to secondary metabolic processes that warranted further investigation.

#### Metabolic shifts between primary and secondary metabolism in response to elevated light exposure throughout berry development

**Figure 8:**
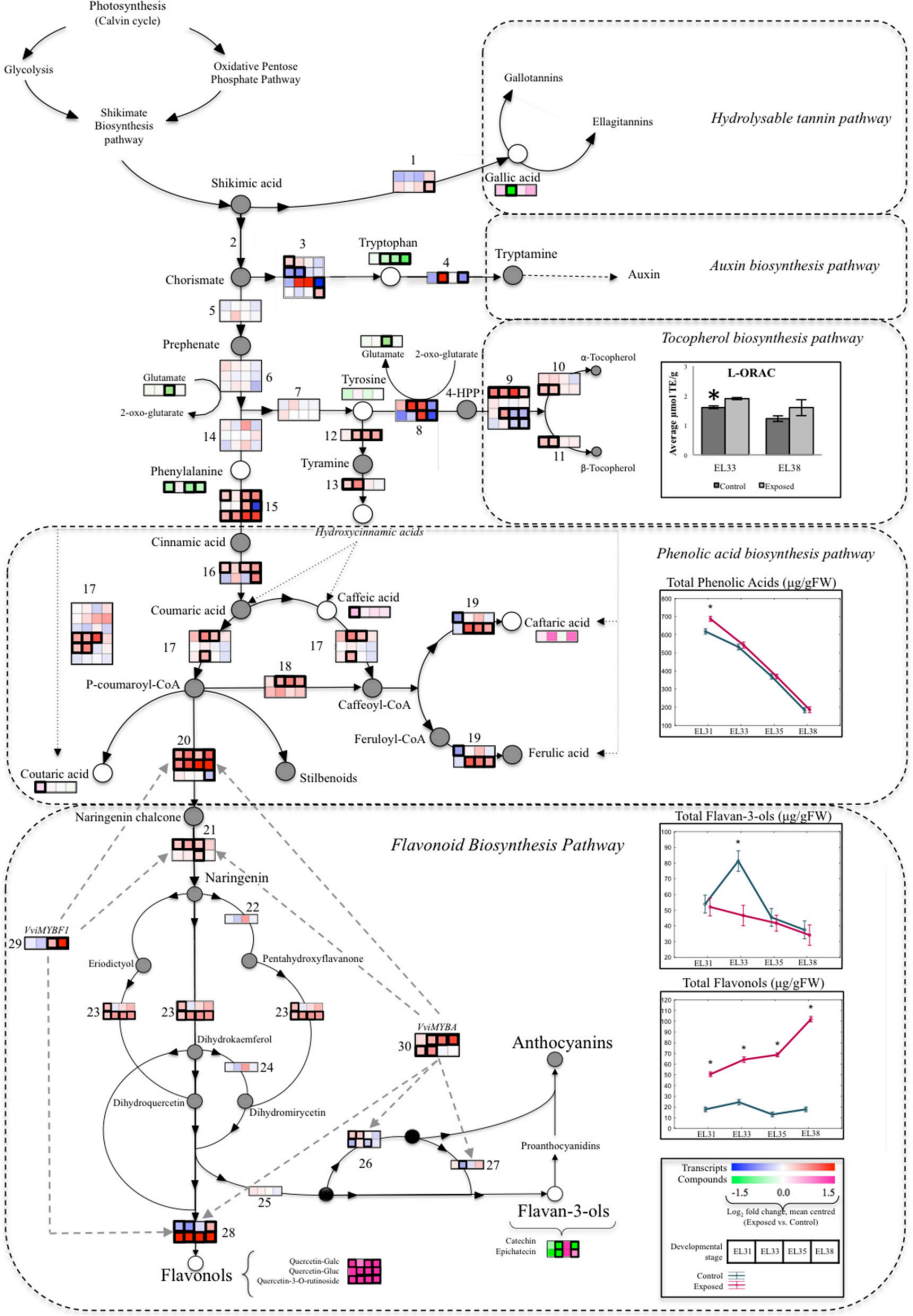
A summarized overview of the branch points between primary berry metabolism toward the phenylpropanoid pathway overlaying transcriptomic and metabolomic data generated from exposed and control grapes harvested at each phenological stage. Blocks indicate the mean-centered log_2_ fold change of the FPKM expression value of the specific transcripts and metabolites involved in the particular enzymatic step at each berry developmental stage when comparing exposed to control samples. Significant differential expression (*q* ≤ 0.05) of genes and compounds are indicated by a bold contour (frame). Total concentrations (μg/g FW) of phenolic acids, flavonols and flavan-3-ols are represented by ANOVA line-plots where significant differences (*q* ≤ 0.05) between exposed and control samples are indicated by an asterisks (^*^). Line graphs representing exposed and control samples are staggered along the x-axis representing the respective developmental stages. Gray circles represented compounds that were not measured, whereas black circles represent various possible compounds at the same enzymatic step. Striped gray arrows represent regulatory steps by associated transcription factors. The genes represented by numbers are listed in Table S10.

For the purpose of determining how elevated light exposure could shift developing grape primary and secondary metabolism, a summarized diagram was constructed to evaluate several metabolic branch points by integrating transcriptomic and metabolomic data generated from the same developing grape berries (Figure 8). The diagram overlays the concentrations of AAs, phenolic acids and flavonoid compounds in developing grapes with the expression levels of the transcripts known to be responsible for the enzymatic steps in the metabolic pathway between primary and secondary metabolism (Table S10). This integrated metabolic pathway focused on the branch point at which Shikimic acid could be either utilized toward the synthesis of hydrolysable tannins or toward the synthesis of chorismate, which serves as substrate for multiple downstream metabolic processes that include the synthesis of auxin from Trp or the synthesis of Tyr or Phe. Tyr in turn serves as a substrate for either the synthesis of the lipophilic antioxidants, tocopherol, or the synthesis of hydroxycinnamic acids from tyramine. Phe on the other hand is an aromatic AA that serves as a precursor for the synthesis of several secondary metabolites such as phenolic acids and flavonoid compounds that could serve as antioxidant molecules under abiotic stress conditions.

The synthesis of higher levels of hydroxycinnamic acids in green grapes were facilitated by both the upregulation of genes encoding the catabolism enzymes of Tyr (VIT_07s0005g04480, VIT_13s0019g04540) and Phe (VIT_06s0004g02620, VIT_08s0040g01710, VIT_13s0019g04460) while upregulation of the same Phe catabolism genes facilitated the accumulation of higher levels of flavonols. The upregulation of a different set of Tyr catabolic enzyme genes (VIT_00s0394g00040, VIT_00s0225g00230, VIT_10s0116g01660, VIT_12s0028g00710, VIT_16s0039g01410) simultaneously contributed to the transcription of tocopherols that subsequently lead to the accumulation of elevated lipophilic antioxidant levels (L-ORAC) in green grapes exposed to elevated light (Figure 8).

## Discussion

Molecular profiling tools provide sensitive and comprehensive snapshots of how a plant/organ/tissue is responding at a specific point in time. It is quite obvious that the value of these molecular snapshots is amplified if they are framed by an accurate understanding of the environmental cues, the developmental stage and general plant status of the plant. This has lead to a renewed focus on integrating accurate measurements of environmental impact factors with grapevine phenotypes observed, specifically in grapevine berries. Several recent studies have advanced our understanding of berry development, ripening and reactions to stress signals and have convincingly shown that berries throughout their growth curve react to their microclimatic environments, but with different responses (Zenoni et al., 2010; Sweetman et al., 2012; Palumbo et al., 2014; Pilati et al., 2014; Wong et al., 2016). Interestingly, many of these studies also showed the resilience of berries to mitigate mild stresses (Carbonell-Bejerano et al., 2013; Martinez-Luscher et al., 2014; Rienth et al., 2014; Wu et al., 2014; Ghan et al., 2015; Suzuki et al., 2015; Joubert et al., 2016; Santo et al., 2016; Savoi et al., 2016; Young et al., 2016; Sun et al., 2017) leading to minimal impacts on overall berry growth and development. How this is orchestrated/managed was the focus of this study, and an experimental system that was previously proven to render grape berries more exposed to light, with minimal changes in berry temperatures, was used (validation of light as the main experimental parameter in the vineyard experiment was described in Young et al., 2016).

### The grape berry developmental profile remained the strongest transcriptional driver despite elevated light exposure

Our data confirmed that development remained the strongest driver for the statistical separation of the grape samples based on their transcriptomes, regardless of viticulture treatment implemented. On average, not more than 8% of the berry transcriptome was affected by the elevated exposure at any of the developmental stages evaluated. As expected, berries in the green developmental stages were transcriptionally more similar in the global sense to each other than to berries from the ripening stages. Developmental phase-specific biomarkers were identified as genes that were responsible for the greatest transcriptional differences observed between green and ripening grape berries. Not only were 48 of the biomarkers identified in this study (Figure S4) also previously established as biomarkers by other research groups (Zamboni et al., 2010; Palumbo et al., 2014), but all, except nine of these genes, were unaffected by elevated light exposure at the stages when the berries were either green, ripening or throughout development.

### Green grapes maintain growth and development by protecting the photosynthetic machinery under light stress conditions

It was previously shown that the exposed grape berries were not different from their control counterparts in terms of size and weight, sugar accumulation and acid degradation patterns (Young et al., 2016) and the transcriptional data also showed that gene expression associated with growth and development, and primary metabolism was not altered by the leaf removal treatment (this study). Despite this fact, photosynthesis-related gene expression, that forms part of primary metabolism, proved to be (the most) significantly affected by the treatment in green grapes.

Our data confirmed that the green berries responded to the increased exposure to try and mitigate the light stress—the first line of defense against potentially damaging effects of photodamage, was the simultaneous activation of several avoidance strategies. One of the strong reactions was the transcription and synthesis of phenolic compounds and tocopherols that were activated, presumably to maintain the redox balance.

Among the phenolic compounds that accumulated at higher levels in response to elevated light were hydroxycinnamic acids and flavonols. Both hydroxycinnamic acids and flavonols can limit photodamage through their ability to scavenge free radicals and ROS, thereby contributing to the maintenance of oxidative homeostasis (Tattini et al., 2005; Agati et al., 2007, 2012, 2013). Flavonols, however, additionally possess the ability to act as sunscreen molecules themselves. They achieve this by absorbing highly energetic solar wavelengths, thereby limiting the generation of ROS due to photooxidation. Although flavonol levels have been found to be negligibly low in developing grape berries, the transcription and subsequent accumulation of these compounds in both a light-dependent and development-independent manner have been reported and extensively characterized in grapes (reviewed by Downey et al., 2006; Czemmel et al., 2009; Matus et al., 2009; Malacarne et al., 2016; Yu et al., 2016; Pastore et al., 2017).

The other avoidance mechanism activated in the exposed berries was non-photochemical quenching, the process by which a large part of excitation energy generated by excessive light exposure can be dissipated as heat (via carotenoids). Through this process, the xanthophyll cycle is activated in which the xanthophyll pigment, violaxanthin, is de-epoxidized to zeaxanthin through the activity of the violaxanthin de-epoxidase (VDE) enzyme, thereby limiting energy transfer from LHCII to PSII. Although the carotenoid metabolites and their ratio's, as well as the transcriptional activation and elevated synthesis of the VDE enzyme already confirmed that the green berries have activated the xanthophyll cycle pigments (Young et al., 2016), the transcriptional mechanism of NPQ activation could be further explored in this study. The PsbS subunit of PSII has been established as the enzyme responsible for “sensing” the impending light stress and initiating NPQ (Li et al., 2000; Gregan and Jordan, 2016). The gene encoding the grapevine PsbS enzyme was found to be significantly upregulated by the leaf removal treatment from the onset of green berry development, potentially linking to the activation and upregulation of the VDE enzyme and subsequent increase of the xanthophyll pool as reported in Young et al. (2016).

However, as high levels of light exposure were maintained throughout the season, it appears that damage to the photosynthetic machinery could no longer be avoided through NPQ alone. At the second green developmental stage (EL33); the process of reversible photoinhibition (RPI) was subsequently activated in an attempt to no longer avoid, but rather acclimate to the continuous light stress, while the synthesis of other antioxidant molecules such as tocopherol and flavonols remained transcriptionally and metabolically upregulated (Figure 8, Table S10). RPI is the process in which photodamage is actively concentrated to the reaction-center binding D1 protein that forms part of Photosystem II (Kyle et al., 1984; Powles, 1984). In doing so, the rapid and ongoing turnover of the D1 protein is ensured through the disorganization of the PSII-LCHII supercomplex in order to remove and replace the damaged D1 protein with a newly synthesized copy. This results in the protection of the photosynthetic machinery from photooxidative stress.

These photoprotective strategies have been well characterized and extensively reported in vegetative tissues (leaves and stems) of numerous plant species (Li et al., 2000; Crouchman et al., 2006; Kato et al., 2012; Niyogi and Truong, 2013; Gorecka et al., 2014). To our knowledge, NPQ and RPI have not been thoroughly investigated in the context of green grape development.

Young et al. (2016) showed higher carotenoid levels (especially xanthophylls) in the exposed berries, yet chlorophyll a: chlorophyll b and total carotene: chlorophyll ratios were maintained in the earlier stages (up until véraison). Total chlorophyll, and the levels of the major photosynthetic carotenoids (β-carotene and lutein) were also not significantly affected. The authors concluded that a pool of carotenoids (predominantly xanthophylls) were responsive to the treatment and increased in response to the increased exposure (light). Since the major carotenoids and chlorophylls were seemingly unaffected, the authors concluded that the increased pool of xanthophylls were able to protect the photosynthetic machinery for normal development to proceed (without damage). The data presented here shows that on a transcriptional level the structural proteins of photosynthesis were significantly upregulated and indicated that there was a higher demand for these proteins possibly due to an increased turnover (damage and repair cycle). Kyzeridou et al. (2015) demonstrated the green fruits of *Nerium oleander* and *Rosa* sp. have a higher cyclic electron flow activity around PSI, when compared to leaves. Kotakis et al. (2006) further showed that cyclic electron flow is enhanced (at the expense of the linear photosynthetic electron flow) in twig collenchyma to adjust potential ATP/NADPH ratios and/or to counteract the detrimental effects of hypoxia. This, combined with the increased activity of non-photochemical quenching via the xanthophyll cycle observed in apple (Cheng and Ma, 2004) and grapevine (Young et al., 2016), suggest that non-foliar photosynthesis is possibly required to produce ATP in organs where gas exchange is prevented (Kalachanis and Manetas, 2010).

In this study, the sequential and simultaneous transcriptional activation of light stress mitigation mechanisms proved to be effective in avoiding irreversible photoinhibition and maintaining the development and growth of grapes. This was evident in the global transcriptional responses and the accumulation of AAs that remained predominantly unaffected by the treatment in the green berries. Furthermore, the AAs considered as oxidative stress markers, Pro and GABA, remained unaffected by the treatment in the green grapes despite elevated exposure to light.

This combination of NPQ, RPI and development-independent flavonol synthesis, although effective in stress mitigation and acclimation, appeared to be energetically costly to the developing green grapes. Firstly, hydroxycinnamic acids were no longer differentially accumulated in response to elevated light, although the transcription and accumulation of flavonols remained dramatically higher in exposed grapes (Figure 8, Table S10). This might be explained by the fact that these compounds compete for the same aromatic AAs, Phe and Tyr, as precursors. Results to this effect were previously reported in tomato leaves exposed to various abiotic stresses (Martinez et al., 2016). The authors demonstrated that flavonols were more effective in the maintenance of oxidative homeostasis than hydroxycinnamic acids when precursors were limited. Furthermore, the MYB transcription factors known to regulate the transcription of several enzymatic steps involved in flavonoid synthesis (Czemmel et al., 2009) were significantly upregulated at each of the developmental stages (Figure 8). Secondly, the upregulation of several AA catabolic enzymes were further testament to the limitations placed on grape berry energetic resources as a consequence of photoprotection. AAs are involved in highly regulated metabolic networks and are crucial for the synthesis of proteins whilst also acting as precursors for a myriad of downstream metabolic processes. AAs have not only been implicated in normal growth and development but also in stress tolerance as their degradation may provide energetic advantage to maintain stress response mechanisms which prove to be energetically expensive to plant metabolism under suboptimal growing conditions. In *Arabidopsis*, evidence exist that transcription of AA catabolic enzymes, with the exception of Pro catabolic enzymes, were more sensitive to abiotic stresses than that of the enzymes responsible for AA synthesis (Less and Galili, 2008). Caldana et al. (2011) showed that amino acid catabolism serves as the main cellular energy supply under adverse environmental conditions as inferred by high-density kinetic analysis. The activity of these catabolic enzymes could therefore provide metabolic energy generated from the breakdown of AAs for utilization toward maintaining plant primary metabolism under stressful biotic and abiotic conditions. Additionally, it has been proposed that excessive accumulation of the branched chain amino acids, or rapid protein turnover induced by adverse environmental conditions could potentially lead to cellular apoptosis as a result of respiratory oxidation. The catabolic breakdown of these AAs is seen as a necessary detoxification mechanism under these conditions, as observed in *Arabidopsis* cell cultures (Taylor, 2004). Since, the branched chain AAs did not accumulate differentially in our investigation (Figure S5) we, however, did not consider it the likely metabolic driver for the differential transcription of AA catabolic enzyme encoding genes.

Genes characterized in one of the aforementioned studies (Less and Galili, 2008) were utilized to identify homologous grapevine genes and their expression analysis in our investigation yielded similar results to previous reports. Transcription of the enzymes responsible for AA synthesis was predominantly unaltered by the elevated light exposure treatment whereas genes encoding the AA catabolic enzymes were far more sensitive to the treatment in comparison (Figure 7).

The catabolism of AAs during the green berry developmental stages therefore could have provided the green grapes with substrates necessary for downstream metabolic reactions when energetically costly abiotic stress protection mechanisms were simultaneously activated. These included the maintenance of nitrogen fixation that lead to slightly shifted substrate utilization and lower accumulation of Asn, Asp, and Gln levels. The accumulation of lower levels of Phe that serves as the precursor for flavonols necessarily synthesized to protect the grapes against elevated light, were also evident, similar to the mechanisms implemented by vegetative plant organs.

Significantly higher concentrations of Gly in response to the light treatment further substantiate the notion that green grapes respond to light stress as vegetative, source organs. Gly and the enzymes responsible for its decarboxylation, Gly decarboxylase complex (GDC) play an integral part in the successful functioning of photorespiration system. Increased photosynthesis and subsequent elevated levels of electron flow through the photosystems as a means to protect the photosynthetic machinery from light stress, is proposed to cause an altered redox state that ultimately influences the rate of photorespiration (Hutchison et al., 2000; Wingler et al., 2000; Voss et al., 2013). Despite elevated expression levels of the GDC encoding genes reported in our investigation (Figure 7A), the GDC themselves are prone to oxidation, hereby causing the accumulation of Gly under high light. Furthermore, Gly is considered to be the rate-determining compound in the synthesis of the antioxidant, glutathione, that might contribute to maintaining the oxidative homeostasis within the developing grape berry. This effect that elevated light exposure had on photorespiration and subsequent high Gly accumulation were previously reported in *Arabidopsis* (Caldana et al., 2011; Florian et al., 2014). To further support this proposed link between Gly and protection of the photosynthetic machinery in green grapes, the difference in the concentration of Gly when comparing exposed to control grapes become less significant as photosynthetic activity declines throughout berry development.

These findings established that green grapes responded to elevated light exposure by activating and refining stress mitigation strategies to predominantly protect the photosynthetic machinery similar to vegetative plant organs. In an attempt to prioritize growth and development, green grapes utilized and combined several precursor substrates and mechanisms to maintain photoprotection and the synthesis of flavonols, regardless of limited energetic resources.

### Ripening berries do not effectively mitigate the effects of light stress

Véraison is the grape developmental stage during which the berry begins to transition from being a photosynthesizing, organ toward becoming a senescing organ while it retains metabolic characteristics of both berry developmental phases on a transcriptional level, as reported here. Véraison has further been extensively characterized by an oxidative burst that includes the production of ROS (particularly H_2_O_2_) that serves as a signaling molecule to signify the initiation of the ripening (Pilati et al., 2007). It would be reasonable to expect that the production of low-levels of H_2_O_2_ as a consequence of light stress along with this developmentally driven oxidative burst could culminate toward a redox imbalance in berries exposed to elevated light. In contrast, the grapes that were exposed to elevated light at véraison did not accumulate higher levels of the known stress markers, Pro and GABA, however, at EL38, when the grapes were no longer photosynthetically active, these stress markers did accumulate at higher levels in exposed grapes. It would therefore be reasonable to speculate that this could be a reflection of the berries' successful limitation of the accumulation of ROS through the combination of NPQ, RPI and flavonol production until véraison (Figures 4, 6, 8).

The rapid accumulation of both Pro and the non-protein AA, GABA, have been extensively reported in plants exposed to abiotic stresses and the metabolism of these AAs are intimately linked (Figure 7). Pro has been shown to enhance primary photochemical activity of thylakoid membranes by limiting photoinhibition and its synthesis is highly sensitive to light (Alia et al., 1997). Furthermore, in grapevine leaves, it has been reported that Pro has the ability to limit inactivation of some antioxidant enzymes while further being capable of stimulating the expression of others (Agudelo-Romero et al., 2013). Therefore, the importance of Pro homeostasis, as opposed to its accumulation, in response to oxidative stress has gained particular interest in the context of plant abiotic stress response (Kavi Kishor and Sreenivasulu, 2014). The homeostasis of Pro levels was found to be imperative to actively dividing plant cells to sustain growth despite exposure to long-term stress. GABA, on the other hand, is capable of either contributing to plant abiotic stress response through its involvement as either a stress signal amplifier or in the maintenance of the carbon: nitrogen ratio under stressful conditions (Barbosa et al., 2010; Kinnersley and Turano, 2010). The accumulation of elevated levels of both Pro and GABA can therefore be associated with plants experiencing abiotic stress symptoms.

Similar to the earlier green developmental stages, the maintenance of photoprotective mechanisms throughout most of the berry development comes at an energetic cost to the grapes that are at this stage no longer accumulating precursors and energy at the rate that photosynthesizing organs are able to. This energetic strain on the grapes are reflected in lower levels of almost half of the AAs measured in these grapes as well as lower total AA concentrations overall measured in the grapes exposed to elevated light.

The transcription and accumulation of flavonols remained elevated in an attempt to protect the berries from light damage and at this stage, the antioxidant pool available to the ripe berries were additionally supplemented by higher levels of apocarotenoid accumulation as reported earlier (Young et al., 2016). Due to significantly higher transcription involved in photosynthesis-related proteins during the early developmental stages, combined with increased carotenoids provides a larger pool of substrates for the degradation via carotenoid cleavage enzymes (CCDs). This leads to an increased apocarotenoid pool in the later stages. Although these compounds are thought of as mere degradation products or volatile impact odorants; they also function as antioxidants and it is speculated that apocarotenoids may play an important signaling role in plant development and in responses to environmental stimuli (Avendaño-Vázquez et al., 2014; Hou et al., 2016).

Similarly, we hypothesize that higher concentrations of several AAs at EL38 (Table 1) in response to elevated light exposure may not be a consequence of transcription of the related biosynthetic enzyme genes at this late developmental stage, but rather due to the systematic degradation of higher protein levels synthesized during early development. The degradation of higher protein levels could therefore liberate higher levels of the respective AA constituents. The dramatic and consistent upregulation of numerous heat shock proteins throughout berry development (Table S7) further supports this hypothesis because of their well-established role as molecular chaperones associated with protein recycling in response to abiotic stress in other plant models as reviewed in Wang et al. (2004).

This systematic shut-down of the protection strategies as the grapes reach maturity were further evident by the fact that the lipophilic antioxidant capacity (L-ORAC) of these grapes were no longer elevated significantly and that Pro and GABA levels were significantly higher in exposed compared to control grapes at this stage. Although the oxidative homeostasis of these grapes were no longer entirely intact (as evident by elevated Pro and GABA levels), it is however important to consider that despite the light-induced stress status of these grapes at EL38, the sole purpose of the fruit had been achieved in the successful development and maturation of the grape seed. The redox-balance and stress responses of the grape berry were no longer of critical importance to the final development of the fruit as evident by the fact that the exposed and control grapes were not physically distinguishable when they were ripe.

## Conclusion

In this study, we aimed to determine how developing Sauvignon blanc grapes manage to maintain primary metabolism and development despite being exposed and responding to non-lethal light stress. Our approach was to explore the global transcriptional response of grapes sampled from a highly characterized vineyard to determine how these grapes acclimated to light stress on a transcriptional level and to elucidate the metabolic consequences of these transcriptional changes. This approach allowed us to demonstrate that a leaf removal treatment in the berry bunch zone of developing Sauvignon blanc grape berries lead to the activation and refinement of several stress avoidance and tolerance strategies in parallel for the purpose of mitigating the effects of light stress whilst maintaining the normal developmental program of the grapes.

These results revealed that photosynthetically active berries are successful at mitigating the effects of light stress much like other vegetative plant organs by potentially limiting the synthesis and distribution of potentially harmful ROS through the continuous turnover of the photosynthetic machinery and the production of light-absorbing flavonoid compounds as well as higher levels of carotenoids in green berries and subsequent apocarotenoids in ripe berries. These grapes achieved a state of acclimation through the redistribution of energy resources in the form of AA catabolism that provided energy precursors and substrates that contributed to the maintenance of these energetically costly stress mitigation mechanisms. To this end, green, photosynthesizing grapes maintain growth and development at all costs to protect the development and maturation of the grape seed.

## Author contributions

MV and PY conceptualized and planned the study. PY implemented and maintained the viticultural treatments and was responsible for the berry sampling. KdP performed RNA processing and RNASeq data analysis. HE performed HPLC analysis for AAs and phenolic compounds. KdP, PY, and MV drafted the original manuscript and all authors contributed and finalized the publication.

### Conflict of interest statement

The authors declare that the research was conducted in the absence of any commercial or financial relationships that could be construed as a potential conflict of interest.

## Supplementary material

Figure S1 Pearson correlation matrix representing the entire transcriptomes of the initial 24 samples representing 3 biological replicates from control and exposed grapes at four phenological stages.

Figure S2 ReviGO analysis output of GO enrichment data generated from the 5050 genes in the grapevine genome that was not expressed whatsoever in the grapes investigated in this study.

Figure S3 Summarized results generated from Real-time PCR analysis.

Figure S4 Venn diagram comparing the molecular biomarkers generated in this study to previously published biomarkers from Zamboni et al. (2010) and Palumbo et al. (2014) and differential expression analysis of biomarkers shared between this investigation and previously published biomarkers.

Figure S5 The amino acid super pathway of Ile, Val, and Leu biosynthesis.

Table S1 Primers used for Real-time PCR.

Table S2 A table summarizing the retention times of phenolic compounds measured.

Table S3 Summary of RNASeq reads and mapping.

Table S4 List of positive and negative molecular biomarkers separating green (EL31 and EL33) from ripening (EL35 and EL38) berries.

Table S5 Table listing the genes most significantly up and downregulated at each developmental stage (−2 > Log_2_FC > 2).

Table S6 Table listing all significantly differentially expressed genes (*q* ≤ 0.05; 1.5 ≤ Log_2_FC ≤ −1.5) significantly correlated to predetermined gene expression clusters according to STEM analysis.

Table S7 Functional annotation (Grimplet et al., 2012) of each of the genes that were highly upregulated (2 ≤ Log_2_FC ≤ −2) between two or more phenological stages indicated in color as represented in Figure 5. *Q-*values represent the level of significant difference between the expression of each indicated gene at the specific developmental stage. Asterisks (^*^) indicate multiple genes represented by the same functional annotation with *Q*-values in this case indicative of the average value of the multiple genes sharing the same function.

Table S8 The amino acid concentrations of all the exposed and control grapes sampled from EL31, EL33, EL35, and EL38. D.N.Q. refers to AA concentrations that were detected but were at concentrations below the limit of quantification.

Table S9 Genes involved in amino acid synthesis and catabolism as indicated by the numbers assigned in Figure 7.

Table S10 The metabolite concentrations and gene expression levels as indicated by the numbers assigned in Figure 8.
