# Supplementary figures and images for: The Transcriptional Responses and Metabolic Consequences of Acclimation to Elevated Light Exposure in Grapevine Berries
Source: Front Plant Sci. 2017 Jul 20;8:1261. doi: 10.3389/fpls.2017.01261 (PMC5518647; doi:10.3389/fpls.2017.01261)

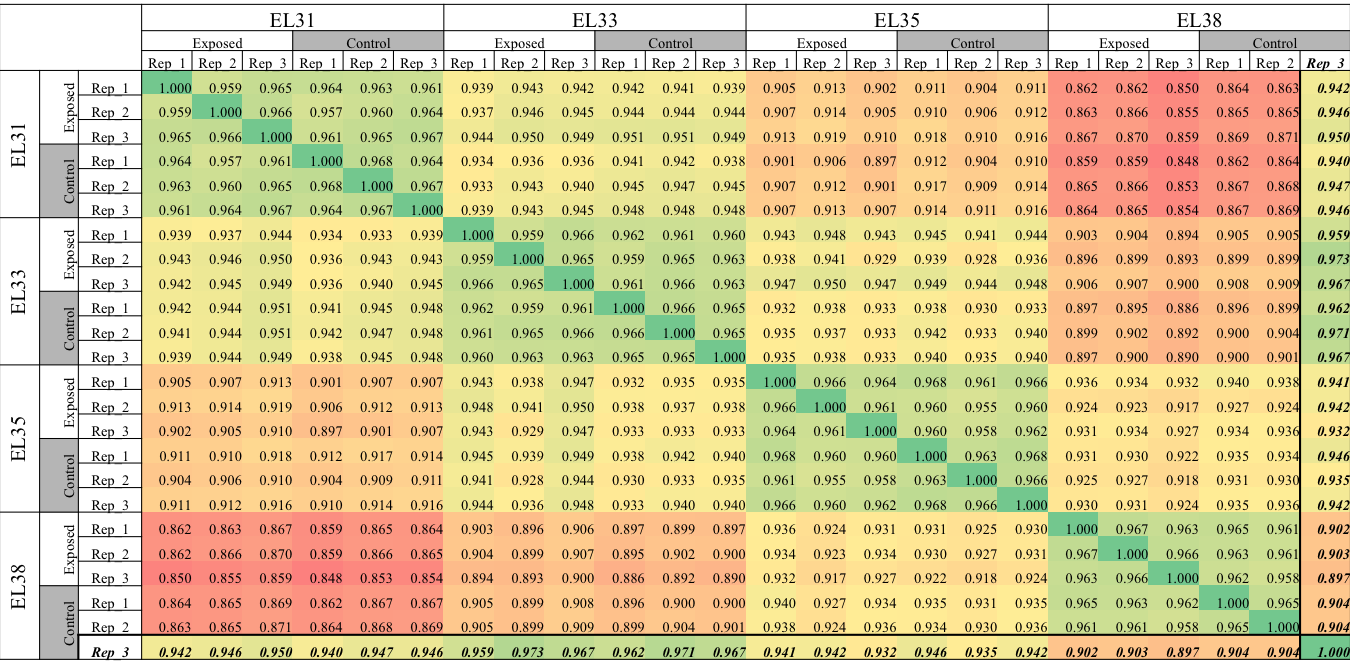

Supplement: Figure S1 — Pearson correlation matrix representing the entire transcriptomes of the initial 24 samples representing 3 biological replicates from control and exposed grapes at four phenological stages. [file Image1.TIFF]

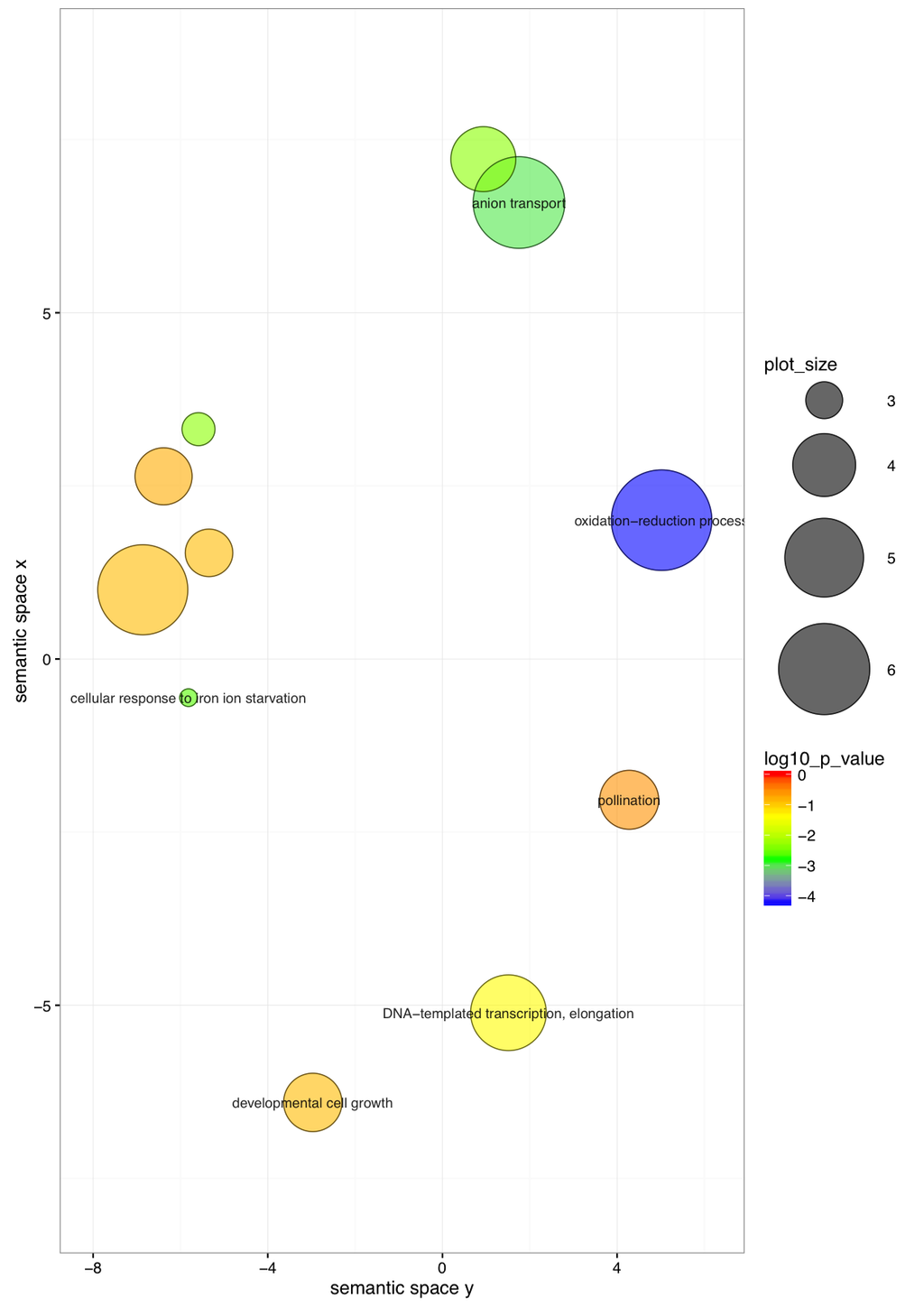

Supplement: Figure S2 — ReviGO analysis output of GO enrichment data generated from the 5050 genes in the grapevine genome that was not expressed whatsoever in the grapes investigated in this study. [file Image2.TIFF]

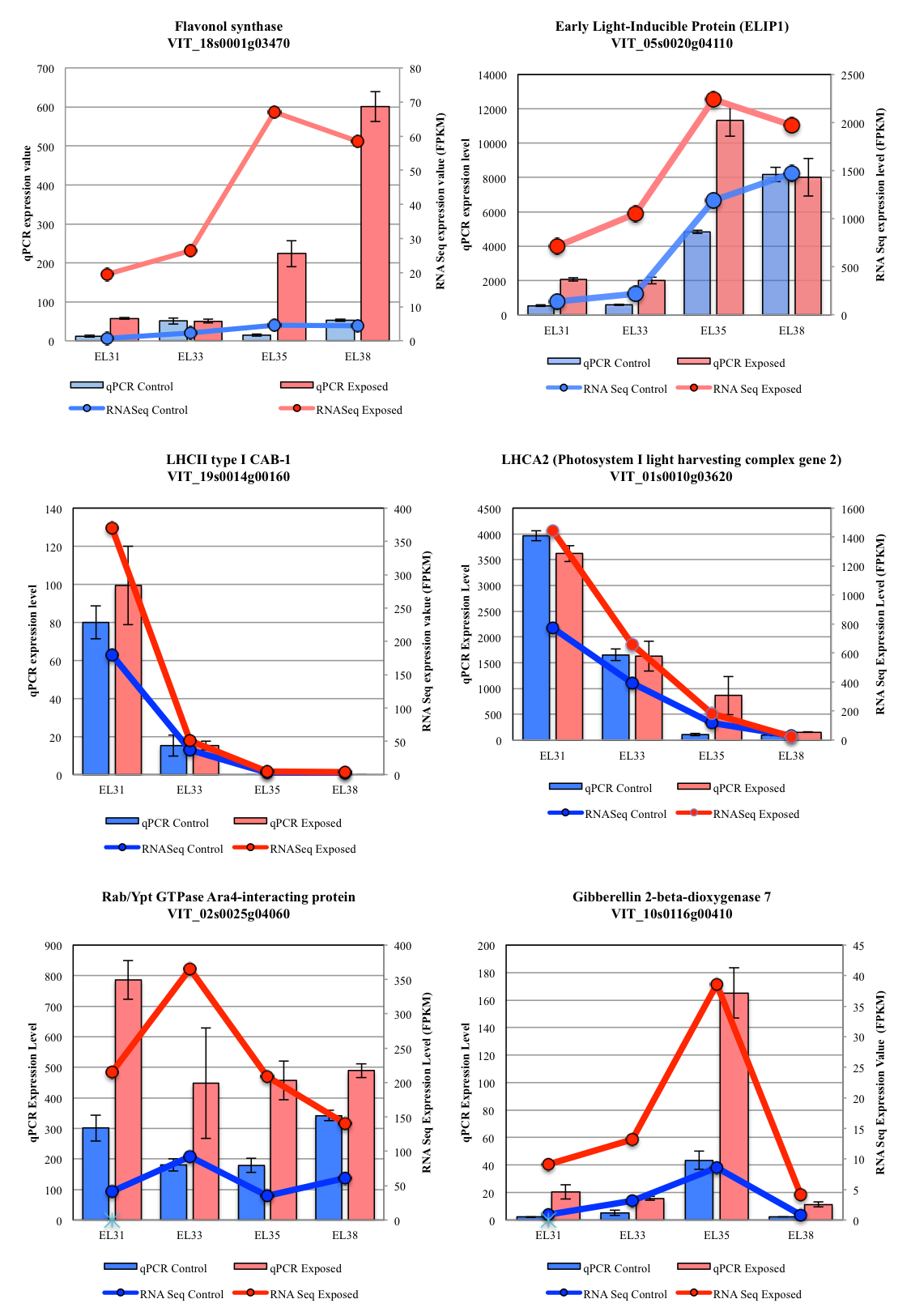

Supplement: Figure S3 — Summarized results generated from Real-time PCR analysis. [file Image3.TIFF]

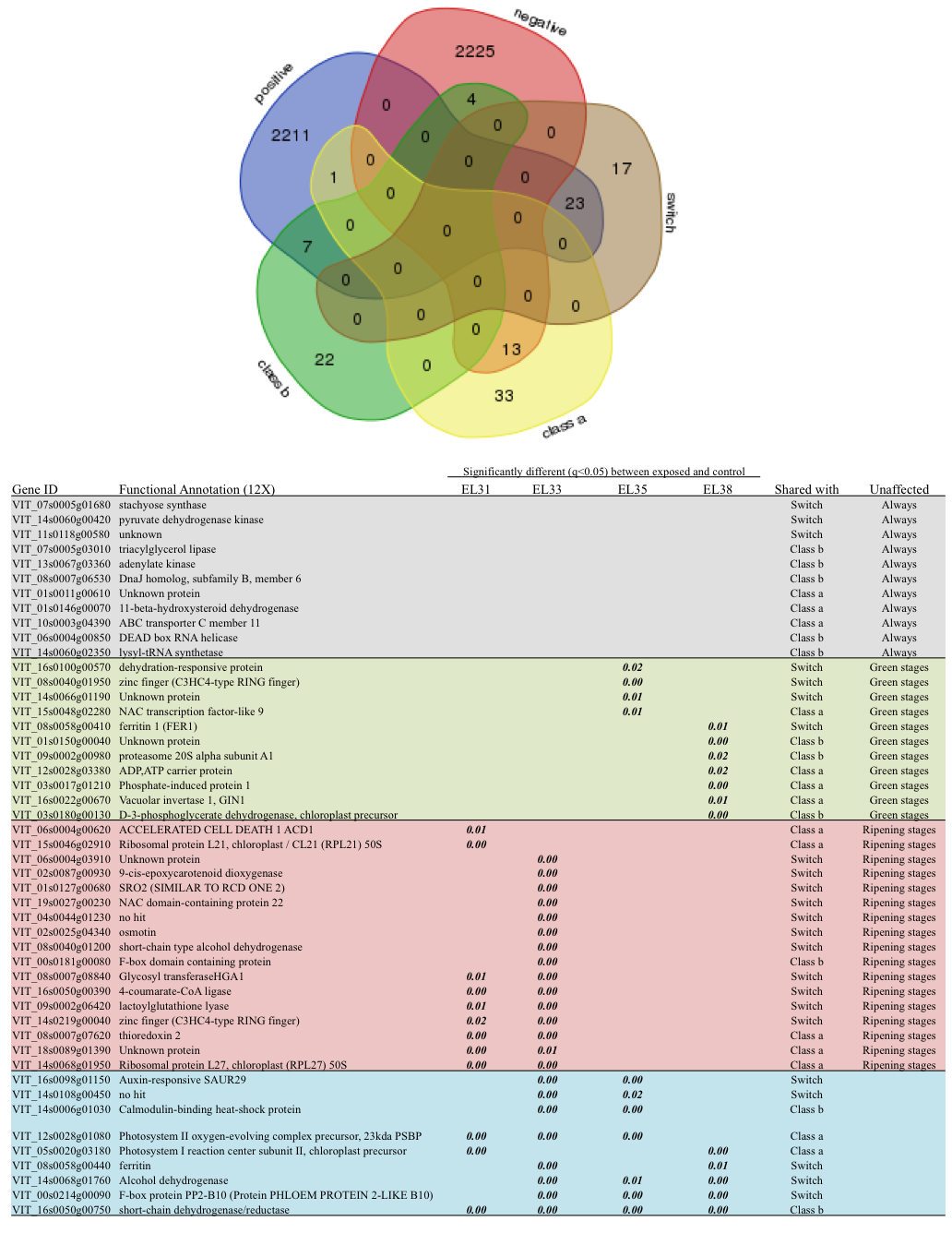

Supplement: Figure S4 — Venn diagram comparing the molecular biomarkers generated in this study to previously published biomarkers from Zamboni et al. (2010) and Palumbo et al. (2014) and differential expression analysis of biomarkers shared between this investigation and previously published biomarkers. [file Image4.TIFF]

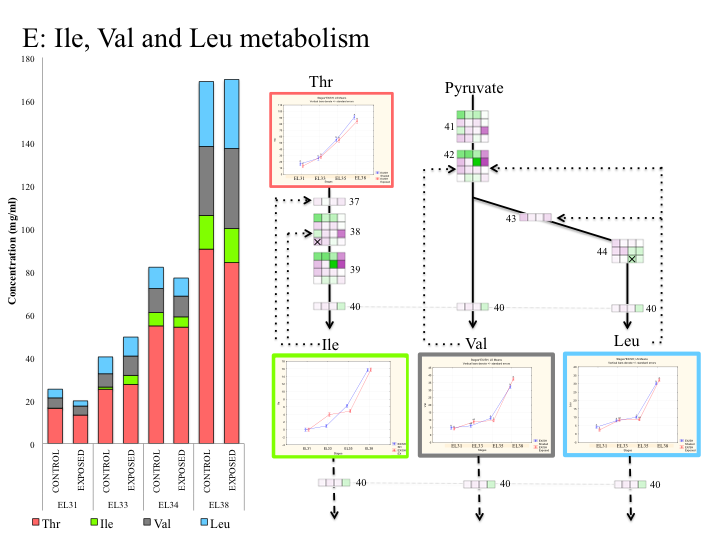

Supplement: Figure S5 — The amino acid super pathway of Ile, Val, and Leu biosynthesis. [file Image5.PNG]
