# Supplementary material for: The Transcriptional Responses and Metabolic Consequences of Acclimation to Elevated Light Exposure in Grapevine Berries
Source: Front Plant Sci. 2017 Jul 20;8:1261. doi: 10.3389/fpls.2017.01261 (PMC5518647; doi:10.3389/fpls.2017.01261)
Supplement: Table S1 — Primers used for Real-time PCR. [file Table1.PDF]

Gene accession	Grimplet Annotation	Primer sequence (5')	Primer sequence (3')	Amplicon length (bp)	Optimal Ta (°C)
VIT_10s0116g00410	gibberellin 2-beta-dioxygenase 7	AGCCAGTTATCGTGGTCCGAAG	TTGTCCCATTGTGGAGCTGAGG	96	63.9615
VIT_18s0001g03470	Flavonol synthase Vitis vinifera	TCCATAACATCTGGCCTCCTCCTG	TCGTTCGCAGCTCTGTAAGTAGG	79	60.6532
VIT_05s0020g04110	ELIP1 (EARLY LIGHT-INDUCABLE PROTEIN)	TGACACGCGTAGCCAACAGAAC	TCGATTTGGCCATCCTCCTTGG	115	65.3547
VIT_02s0025g04060	Rab/Ypt GTPase Ara4-interacting protein	CAGTGAAGGAGATCGAACCATTGC	GCCATATCAATCATGTCCGTGAGC	69	60.9634
VIT_01s0010g03620	LHCA2 (Photosystem I light harvesting complex gene 2)	TTGCCACCATCTCTCCCAGTTC	CCGTGAACTTCTTCAGGCTCAGTC	102	63.5269
VIT_19s0014g00160	LHCII type I CAB-1	TGGACCGCTAGGAGAAGTGAAG	ACCCGAACATGGAGAACATAGCC	65	59.5942
