# Supplementary material for: The Transcriptional Responses and Metabolic Consequences of Acclimation to Elevated Light Exposure in Grapevine Berries
Source: Front Plant Sci. 2017 Jul 20;8:1261. doi: 10.3389/fpls.2017.01261 (PMC5518647; doi:10.3389/fpls.2017.01261)
Supplement: Table S2 — A table summarizing the retention times of phenolic compounds measured. [file Table2.PDF]

	Standards	Rt (min)	Range (mg/L)	Slope	y-Intercept	r2	LOD (g/L)	LOQ (g/L)
**Flavan-3-ols**	Catechin	15.17	263-0	11377.214	-24.146	0.99816	0.016	0.054
	Epicatechin	22.45	107-0	14006.536	-63.675	0.99786	0.009	0.029
**Phenolic acids**	Caftaric acid	13.36	125-0	42534.569	-5.3	0.99976	0.003	0.008
	Caffeic acid	17.4	480-0	102285.985	207.618	0.99938	0.015	0.049
**Flavonols**	Quercetin-Glucoside	40.41	125-1	45538.719	-19.845	0.99773	0.005	0.014
