# Supplementary material for: The Transcriptional Responses and Metabolic Consequences of Acclimation to Elevated Light Exposure in Grapevine Berries
Source: Front Plant Sci. 2017 Jul 20;8:1261. doi: 10.3389/fpls.2017.01261 (PMC5518647; doi:10.3389/fpls.2017.01261)
Supplement: Table S3 — Summary of RNASeq reads and mapping. [file Table3.PDF]

	EL31		EL33		EL35		EL38	
**Treatment**	Control	Exposed	Control	Exposed	Control	Exposed	Control	Exposed
**No. of total reads**	100712837	101310946	130713888	101080327	81231782	77648208	84861214	86362444
**Average overall mapping rate (%)**	88.1	88.2	87.8	87.9	87.2	88	87.1	86.6
**Number of trancripts expressed (VIT_)**	22770	22681	22647	22206	21484	21612	21417	22001
**Number of trancripts represented in V1**	20995	20905	20834	20495	19897	20030	19819	20297
