# Supplementary material for: The Transcriptional Responses and Metabolic Consequences of Acclimation to Elevated Light Exposure in Grapevine Berries
Source: Front Plant Sci. 2017 Jul 20;8:1261. doi: 10.3389/fpls.2017.01261 (PMC5518647; doi:10.3389/fpls.2017.01261)
Supplement: Table S7 — Functional annotation (Grimplet et al., 2012) of each of the genes that were highly upregulated (2 ≤ Log2FC ≤ −2) between two or more phenological stages indicated in color as represented in Figure 5. Q-values represent the level of significant difference between the expression of each indicated gene at the specific developmental stage. Asterisks (*) indicate multiple genes represented by the same functional annotation with Q-values in this case indicative of the average value of the multiple genes sharing the same function. [file Table7.PDF]

Phenological stages	Functional annotation	*Q*-value			
		EL31	EL33	EL35	EL38
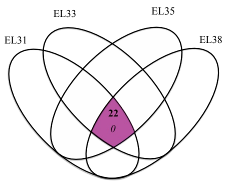	Chaperone	<0.001	<0.001	<0.001	0.042
	Flavonol synthase	<0.001	<0.001	<0.001	<0.001
	Galactinol synthase	<0.001	<0.001	<0.001	<0.001
	Gibberellin 2-beta-dioxygenase 7	<0.001	<0.001	<0.001	<0.001
	Glycosyl transferase family 8 protein	<0.001	<0.001	<0.001	<0.001
	Heat shock protein 17*	<0.001	<0.001	<0.001	<0.001
	Heat shock protein 18*	<0.001	<0.001	<0.001	<0.001
	Heat shock protein 21	<0.001	<0.001	<0.001	<0.001
	Heat shock protein 23*	<0.001	<0.001	<0.001	<0.001
	Heat shock protein 70	<0.001	<0.001	<0.001	<0.001
	Heat shock protein*	<0.001	<0.001	<0.001	<0.001
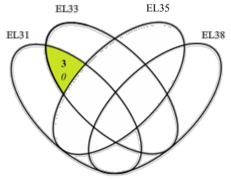	Steroid 5alpha-reductase	<0.001	<0.001	<0.001	0.007
	ELIP1 (Early light inducible protein)	<0.001	<0.001	<0.001	0.016
	Heat shock protein 18	0.008	<0.001	0.268	0.351
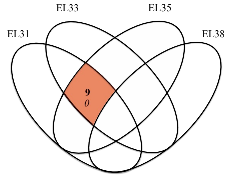	Calmodulin	<0.001	<0.001	<0.001	<0.001
	Heat shock protein 17*	<0.001	<0.001	<0.001	<0.001
	Heat shock protein 18	<0.001	<0.001	<0.001	<0.001
	Heat shock protein 20	<0.001	<0.001	<0.001	<0.001
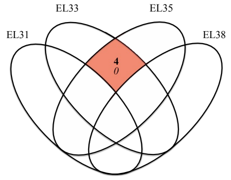	Heat shock protein 17	0.055	<0.001	<0.001	0.091
	Seed maturation protein PM31	0.233	<0.001	<0.001	0.005
	Exocyst subunit EXO70 H2	<0.001	<0.001	<0.001	<0.001
	Ubiquitin-conjugating enzyme E2 variant	0.233	<0.001	<0.001	0.141
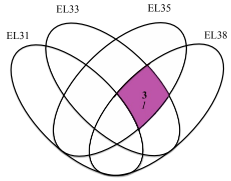	Heat shock protein 21	<0.001	<0.001	<0.001	0.005
	FtsH protease	0.322	<0.001	<0.001	<0.001
	Heat shock protein 17	0.082	<0.001	<0.001	<0.001
	Pentatricopeptide (PPR) repeat-containing	*0.277*	*0.004*	*0.015*	*0.007*
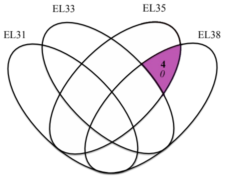	Serine protease inhibitor	1.000	1.000	0.004	0.007
	Aminopeptidase	1.000	1.000	0.004	0.012
	Heat shock protein 16.9	1.000	0.316	<0.001	0.036
	ATAN11 (ANTHOCYANIN11)	0.081	0.829	0.003	<0.001
